# CK1BP Reduces α-Synuclein Oligomerization and Aggregation Independent of Serine 129 Phosphorylation

**DOI:** 10.3390/cells10112830

**Published:** 2021-10-21

**Authors:** Lea Elsholz, Yasmine Wasser, Patrick Ziegler, Pardes Habib, Aaron Voigt

**Affiliations:** 1 Department of Neurology, Medical Faculty, RWTH Aachen University, 52074 Aachen, Germany; lea.elsholz@rwth-aachen.de; 2 Neurobiological Research, University Medical Center and Institute for Biology II, RWTH Aachen University, 52074 Aachen, Germany; yasmine.wasser@rwth-aachen.de; 3 Institute for Occupational, Social and Environmental Medicine, RWTH Aachen University, 52074 Aachen, Germany; pziegler@ukaachen.de; 4 JARA-BRAIN Institute of Molecular Neuroscience and Neuroimaging, Forschungszentrum Jülich GmbH and RWTH Aachen University, 52074 Aachen, Germany

**Keywords:** CK1BP, CK1, α-Synuclein, α-Synuclein oligomerization, α-Synuclein aggregation, Parkinson’s disease, serine 129 phosphorylation, BiFC

## Abstract

The pathological accumulation of α-Synuclein (α-Syn) is the hallmark of neurodegenerative α-synucleinopathies, including Parkinsons’s disease (PD). In contrast to the mostly non-phosphorylated soluble α-Syn, aggregated α-Syn is usually phosphorylated at serine 129 (S129). Therefore, S129-phosphorylation is suspected to interfere with α-Syn aggregation. Among other kinases, protein kinase CK1 (CK1) is known to phosphorylate α-Syn at S129. We overexpressed CK1 binding protein (CK1BP) to inhibit CK1 kinase activity. Using Bimolecular Fluorescence Complementation (BiFC) in combination with biochemical methods, we monitored the S129 phosphorylation and oligomerization of α-Syn in HEK293T cells. We found that CK1BP reduced the overall protein levels of α-Syn. Moreover, CK1BP concomitantly reduced S129 phosphorylation, oligomerization and the amount of insoluble α-Syn. Analyzing different α-Syn variants including S129 mutations, we show that the effects of CK1BP on α-Syn accumulation were independent of S129 phosphorylation. Further analysis of an aggregating polyglutamine (polyQ) protein confirmed a phosphorylation-independent decrease in aggregation. Our results imply that the inhibition of CK1 activity by CK1BP might exert beneficial effects on NDDs in general. Accordingly, CK1BP represents a promising target for the rational design of therapeutic approaches to cease or at least delay the progression of α-synucleinopathies.

## 1. Introduction

Abnormal protein deposition in the central nervous system (CNS) is a common pathologic feature of many neurodegenerative diseases (NDDs). α-Synuclein (α-Syn) is a soluble, presynaptic protein that is found abundantly in neuronal presynapses throughout the brain [1]. Aggregated α-Syn represents the pathological hallmark of so-called α-synucleinopathies. These include Parkinson’s disease (PD), dementia with Lewy bodies (DLB) and multiple system atrophy (MSA) [2,3]. α-Syn abundance appears to be causatively linked to these pathologies. For example, certain mutations in the α-Syn-encoding *SNCA* gene or *SNCA* duplication/triplication cause familial PD [4,5,6,7]. In addition, the presence of insoluble α-Syn correlates with disease in α-synucleinopathies [8,9,10]. Still, the presence of α-Syn inclusions, so-called Lewy bodies (LBs), is not necessarily associated with neurodegeneration in the human brain [11]. Moreover, several studies reported a dissociation between the aggregation and toxicity of α-Syn [12,13,14]. In the course of α-Syn aggregation, a variety of oligomeric species form that differ in structure and solubility, comprising potential precursors of aggregated α-Syn [15,16]. Simultaneously, α-Syn oligomers are specifically increased in diseased brains [15,17,18,19] and displayed neurotoxicity in various research models [20,21,22], underscoring their pathogenic relevance. Thus, identifying the modulators of α-Syn oligomerization and aggregation is crucial to improve our understanding of α-Syn-related neurodegeneration.

Phosphorylation at serine 129 (S129) is the major modification of α-Syn found in LBs, though there is conflicting evidence on how it relates to α-Syn accumulation in NDDs [8,23]. For example, S129 phosphorylation seemed to enhance α-Syn aggregation and altered α-Syn fibril morphology in some studies [9,24]. In contrast, others suggested that S129 phosphorylation was not responsible for the increased aggregation of α-Syn [25,26]. Over the years, a number of kinases have been reported to phosphorylate α-Syn at S129, including members of the polo-like kinase (PLK) and G-protein coupled receptor kinase (GRK) families as well as protein kinases CK1 and CK2 [27,28,29,30]. CK1 is an ubiquitously expressed Ser/Thr kinase involved in several intracellular signaling pathways. In particular, six genetically different human isoforms have been described, named CK1α, γ1-3, δ and ϵ. While all six isoforms show highly homologous kinase domains, they differ in the C-terminal regulatory region. In general, CK1 has been the subject of oncological research. Furthermore, the isoforms CK1δ and CK1ϵ are involved in the regulation of the circadian rhythm (for a detailed review, see [31]) and have been linked to Alzheimer’s disease as well as α-Syn pathology [32,33,34,35]. However, the role and regulation of CK1 kinase activity in α-synucleinopathies is still unclear. Therefore, we used the CK1 binding protein (CK1BP) to study the impact of CK1 kinase activity on the S129 phosphorylation and accumulation of α-Syn.

CK1BP is a 27.8 kDa protein that is found under various names in the literature. CK1BP is also referred to as uncharacterized hypothalamus protein 1 (HSMNP1) or C20orf35 according to the gene locus. Furthermore, the protein was named dysbindin-containing domain 2 (DBNDD2) as CK1BP shares sequence homology with the acidic C-terminal domain of dysbindin [36]. To date, very little is known about the cellular functions of CK1BP. The limited available data on CK1BP suggest a potential relevance of CK1BP expression for apoptosis induction in hematopoietic stem cells or place it in the context of colorectal cancer [37,38,39]. More importantly, CK1BP is highly expressed in human brain and binds to CK1 δ and ϵ isoforms in close proximity to the catalytic domain [36,40]. In 2006, Yin et al. showed that CK1BP thereby inhibits CK1 kinase activity and subsequent substrate phosphorylation in a dose-dependent manner without affecting CK1 protein levels [36].

**Figure A1:**
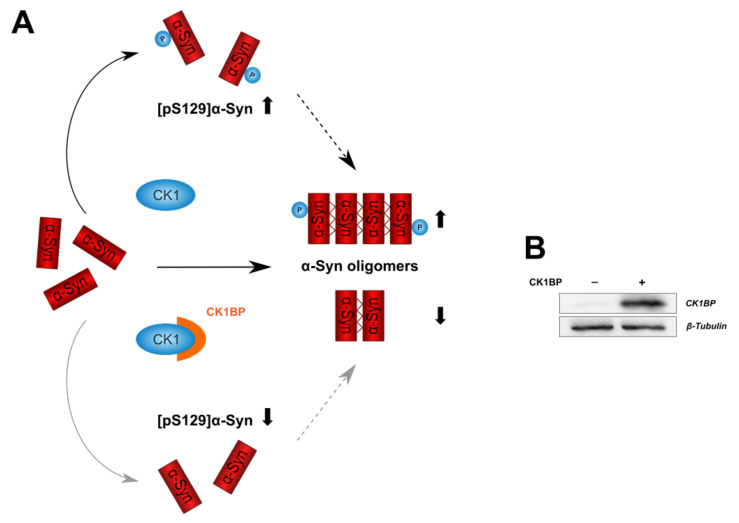
CK1BP overexpression in HEK cells. (**A**) Schematic illustration of a potential link between inhibition of CK1 by CK1BP, S129 phosphorylation of α-Syn and intracellular α-Syn accumulation. CK1 phosphorylates α-Syn at S129. S129 phosphorylation might alter propensity of α-Syn to aggregate. By binding to CK1, CK1BP might affect S129 phosphorylation and multimerization of α-Syn. (**B**) Representative immunoblot displaying endogenous (−) and overexpressed (+) protein levels of CK1BP in the RIPA-soluble protein fraction of HEK cells.

In vitro, CK1BP impaired the CK1-mediated phosphorylation of α-Syn [36]. As S129 phosphorylation might alter the aggregation propensity of α-Syn, we therefore hypothesized that the overexpression of CK1BP might reduce S129 phosphorylation and decrease α-Syn accumulation in a cellular context (Appendix A, Figure A1).

Here, we show that CK1BP indeed reduced S129 phosphorylation and α-Syn oligomerization in our model. However, the observed decrease in α-Syn protein levels was independent of S129 phosphorylation as examined by the expression of mutated α-Syn variants that prevented phosphorylation at position 129. Moreover, CK1BP also decreased the aggregation of proteins other than α-Syn. In summary, we show that CK1BP reduced the oligomerization of disease-associated aggregating proteins in a cellular model.

## 2. Materials and Methods

### 2.1. Cell Culture and Transfection

Human embryonic kidney 293T (HEK) cells (HEK293T; Leibniz Institute DSMZ-German Collection of Microorganisms and Cell Cultures GmbH, Braunschweig, Germany) were cultured at 37 °C and 5% CO${}_{2}$ in Dulbecco’s Modified Eagle Medium (DMEM; PAN-Biotech, Aidenbach, Germany) supplemented with 10% fetal bovine serum (FBS; PAN-Biotech, Aidenbach, Germany) and 0.5% Penicillin/Streptomycin (PAN-Biotech; Aidenbach, Germany).

For transient transfection, cells were seeded with $3.2\times 10^{4}$ cells per cm${}^{2}$. After 24 h, cells were supplemented with fresh medium and transfected with equal amounts of DNA using Metafectene (Biontex Laboratories, München/Laim, Germany) according to the manufacturer’s instructions. Subsequently, cells were cultured for 48 h before further analysis.

α-Syn constructs for Bimolecular Fluorescence Complementation assays (BiFC-α-Syn) were co-transfected as a pair of fusion proteins consisting of the respective α-Syn variant tagged with a non-functional half of the Venus fluorophore on either the N- (VN-α-Syn) or the C-terminus (α-SynVC) [41].

### 2.2. Cell Lysis and Sample Preparation

Forty-eight hours post-transfection, cells were washed thrice with PBS (PAN-Biotech, Aidenbach, Germany) and resuspended in Radio-Immunoprecipitation (RIPA) lysis buffer (pH = 8, 50 mM Tris, 0.1% SDS, 150 mM NaCl, 1% TritonX-100, 0.5% sodium deoxycholate, protease inhibitor cocktail (complete EDTA-free, Roche, Mannheim, Germany)). Cells were incubated for 30 min on ice and cell lysates were centrifuged at 17,000× *g* for 20 min at 4 °C for protein fractionization. The supernatant was collected as RIPA-soluble protein fraction and stored at −20 °C until further use. All following steps were performed at 4 °C. The RIPA-insoluble pellet was washed with RIPA under agitation for 30 min, followed by centrifugation at 17,000× *g* for 30 min. The supernatant was discarded and the remaining pellet was dissolved in UTC buffer (pH = 8.5, 30 mM Tris, 2 M thiourea, 7 M urea, 4% CHAPS). After 1 h of incubation under agitation, samples were sonicated for 10 min and centrifuged for 30 min at 17,000× *g*. The supernatant containing the urea-soluble protein fraction (=RIPA-insoluble protein fraction) of the cell lysates was collected and stored at −20 °C until further use.

### 2.3. Western Blot

Protein concentrations of RIPA-soluble cell lysates were determined using the DC Protein Assay Kit (Bio-Rad, Feldkirchen, Germany) following the manufacturer’s instructions. An amount of 15–30 µg of protein was volume-adjusted, substituted (5:1) with 5× Laemmli buffer (pH = 6.8, 250 mM Tris-HCl, 10% SDS, 1.25% bromophenol blue, 10 mM EDTA, 0.03% β-mercaptoethanol, 50% glycerol) and heated at 95 °C for 5 min. For RIPA-insoluble protein fractions, equal amounts of urea-soluble probes were loaded relative to the protein concentration of the respective RIPA-soluble sample fraction. Samples were loaded on a 12% SDS-polyacrylamide gel and size separation was performed at 100 V for 130 min. Separated proteins were transferred to a nitrocellulose membrane (0.2 µm pore size, GE Healthcare Life Science, Marlborough, MA, USA) by semi-dry transfer. The membrane was washed thrice for 5 min in 1× Tris-buffered saline (pH = 7.5, 25 mM Tris-HCl, 140 mM NaCl) with 0.05% Tween20 (TBS-T) and blocked for 1h at room temperature (RT) with 5% skim milk (Carl Roth, Karlsruhe, Germany) in TBS-T. The membrane was incubated overnight with primary antibody in TBS-T at 4 °C. Primary antibodies used were rabbit anti-pS129-α-Syn (pSer129; 1:2000; Abcam #ab51253, Cambridge, UK), mouse anti-α-Syn (1:2000; BD Transduction Laboratories #BD610786, Franklin Lakes, NJ, USA), mouse anti-polyQ (polyglutamine; 1:2000; Merck Millipore #MAB1574, Darmstadt, Germany) and rabbit anti-CK1BP (DBNDD2; 1:2000; Proteintech #27623-1-AP, Rosemont, IL, USA). Mouse anti-β-Tubulin (E7-s; 1:2000; Developmental Studies Hybridoma Bank, Iowa City, IA, USA) or mouse anti-Actin (ACTN05; 1:2000; Abcam #ab3280, Cambridge, UK) was used for normalization of RIPA-soluble samples. All primary antibodies were kept in TBS-T with 0.02% sodium azide or 2.5% skim milk (pSer129). After incubation with primary antibodies, membranes were washed in TBS-T three times for at least 5 min and incubated for 1 h at RT with respective secondary antibodies (sheep anti-mouse (1:10,000; GE Healthcare #NXA931V, Little Chalfont, Buckinghamshire, UK) or donkey anti-rabbit (1:10,000; GE Healthcare #NA934V, Little Chalfont, Buckinghamshire, UK)) coupled to horse-radish peroxidase (HRP). After washing membranes thrice for at least 5 min in TBS-T, HRP-signal was detected via ECL SuperSignal (Thermo Fisher Scientific, Waltham, MA, USA) in combination with a chemiluminescence detection apparatus (Alliance LD4.77WL.Auto; Biometra, Göttingen, Germany). Measurement of immunoblot band intensities for assessment of protein levels was performed with ImageJ Software (ImageJ 1.53a bundled with Java 1.8.0_112, National Institutes of Health, Bethesda, MD, USA). Intensities of protein bands from Western Blot membrane images were determined using the option “mean gray value” in ImageJ measurement settings. For soluble protein fractions, the indicated intensities of specific protein bands (α-Syn, polyQ) were normalized to the respective loading control (intensity of the same lanE′s protein band for β-tubulin). Intensities of protein bands from the urea fractions were not normalized to housekeeping proteins (β-tubulin, α-Actin etc.) because these proteins do not aggregate and were not stably detectable in the insoluble protein fraction (data not shown). As reported by others, C-terminally tagged BiFC-α-Syn constructs (α-SynVC) showed reduced stability and detection varied between samples compared to N-terminally tagged α-Syn constructs (VN-α-Syn) [42]. For better comparison between samples and experiments, only protein levels of VN-α-Syn (corresponding to 37 kDa band) were shown and quantified for all BiFC-α-Syn variants.

### 2.4. Fluorescence Microscopy

Cells were seeded on cover slips and transfected as described above. After fixation with 4% PFA in PBS for 10 min, cells were blocked and permeabilized simultaneously in PBS with 2% bovine serum albumin (Albumin Fraction V; Roth, Karlsruhe, Germany) and 1% TritonX-100 for 1 h. Cover slips were washed thrice with PBS. DNA was stained with DAPI (1:200 in blocking solution; Carl Roth, Karlsruhe, Germany) for 1 min, followed by three washing steps and F-actin staining with Alexa Fluor 568 Phalloidin (1:200 in blocking solution; Invitrogen, Thermo Fisher Scientific, Waltham, MA, USA) for 20 min. Cover slips were washed two times with PBS and once with dH2O. Cells were mounted in FluoromountG (Southern Biotech, Birmingham, AL, USA) and imaged using an epifluorescence microscope (20× and 40× objectives of Olympus BX51TRF, Tokio, Japan). Similar microscope settings were used for all conditions of the same experiment. Images were recorded with a digital camera (Olympus DP72, Tokio, Japan) and cell⌃F imaging software (Olympus Soft Imaging Solutions, Münster, Germany). ImageJ software (ImageJ 1.53a bundled with Java 1.8.0_112, National Institutes of Health, Bethesda, MD, USA) was used for image processing and analysis.

### 2.5. Flow Cytometry and FACS Analysis

Forty-eight hours after transient transfection, cells were washed with PBS, trypsinized (Trypsin/EDTA, PAN-Biotech, Aidenbach, Germany) for 5 min and centrifuged in cold culture medium for 5 min at 400× *g*. After washing with FACS buffer (2% FBS, 2 mM EDTA in PBS), cell pellet was resolved in FACS buffer and directly subjected to flow cytometry or fixed in 2% PFA in PBS and stored for up to 24 h until FACS analysis at a FACSCanto${}^{\mathrm{TM}}$ II (BD Transduction Laboratories, Franklin Lakes, NJ, USA). Forward and side scatter signals were used to restrict the analysis to viable cells. Venus/GFP fluorescence intensity (FL1, *x*-axis) was plotted on a log scale against FL4 (*y*-axis), which was not used for the experiments. Signal amplification was set so that background fluorescence of non-expressing cells was below 100. In total, 50,000 cells per sample were acquired. Data were processed using FlowJo software (Treestar Inc., Ashland, OR, USA). Fluorescence measurement is presented as a one-dimensional histogram. Cells were gated based on signal intensities and the number of cells in each population was counted separately. A GFP-transfected positive control condition served as template for the identification of fluorescent cells in every experiment. Fluorescent cell fractions (FCFs) were indicated as proportion of fluorescent cells of each sample and included cells with a fluorescence intensity of <10${}^{3}$ (low FCF), $10^{3}$–$10^{4}$ (medium FCF) and >10${}^{4}$ (high FCF) on a logarithmic scale.

### 2.6. Statistical Analysis

Data analysis was carried out using GraphPad Prism software (GraphPad Prism version 8.0.2 for Windows, GraphPad Software, San Diego, CA, USA). Outliers in Western Blot band intensity quantification datasets were excluded, as identified by ROUT method. Differences in the percentages of fluorescent cells between CK1BP and the respective control groups were determined with one-tailed Wilcoxon matched-pairs signed rank test. Differences in FCFs between conditions of CK1BP co-expression and the matched control groups were determined with two-way ANOVA followed by Tukey’s multiple comparisons test. Values of p<0.05 were considered significant. The number of experimental replicates and the statistical tests applied are indicated in the respective figure legends.

## 3. Results

### 3.1. Monitoring Intracellular α-Syn Oligomerization with BiFC

Human embryonic kidney 293T (HEK) cells were reported to abundantly express endogenous protein kinase CK1 (CK1) [36]. Moreover, endogenous α-Syn protein levels in HEK cells are not detectable without the special treatment of Western Blot membranes [43] (see Supplementary Materials). Thus, HEK cells form a controlled cellular environment without endogenous α-Syn that would obscure subsequent analyses. Accordingly, HEK cells provide an ideal tool to assess the impact of changes in CK1 kinase activity on α-Syn S129 phosphorylation and α-Syn aggregation propensity.

**Figure A2:**
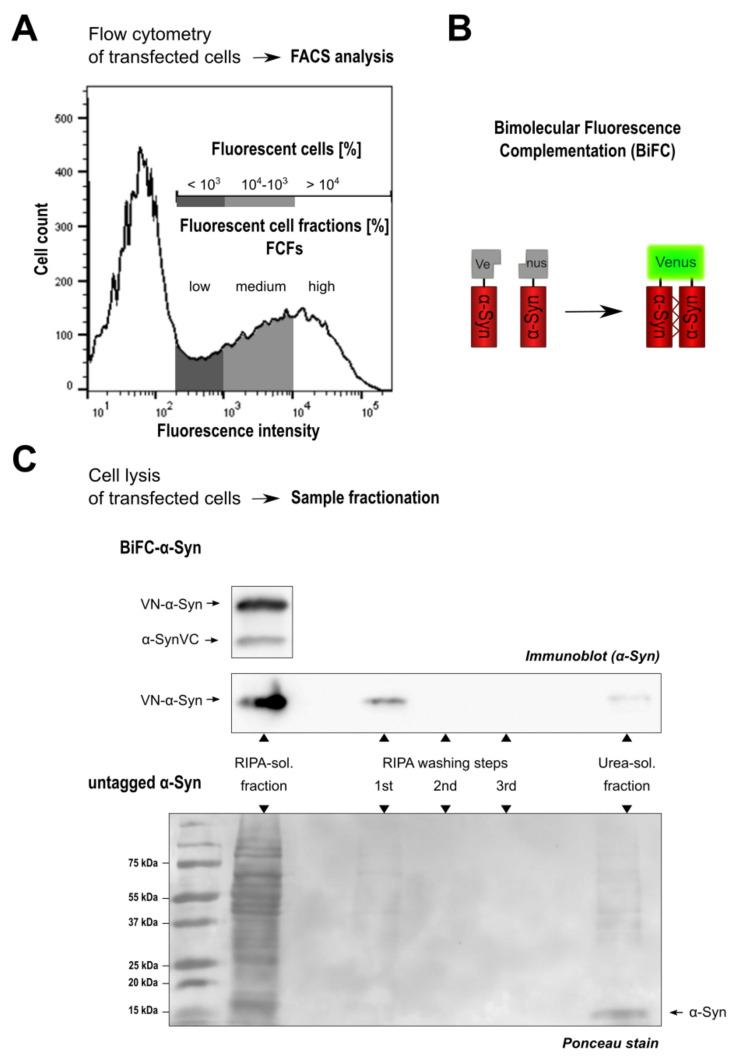
Sample preparation and analysis of α-Syn multimerization and solubility. FACS analysis of fluorescent cells and sample fractionation of cell lysates were used to assess the abundance of oligomeric, soluble and insoluble α-Syn. (**A**) Representative histogram of HEK cells transfected with GFP. FACS analysis of GFP-transfected cells served as template to identify the percentage of fluorescent cells in all samples of the corresponding experiment. Fluorescent cells were categorized into three fluorescent cell fractions (FCFs) according to fluorescence intensity. Cells are represented as follows: fluorescence intensities <$10^{3}$ in the low FCF (dark grey), fluorescence intensities $10^{3}$–$10^{4}$ in the medium FCF (light grey) and fluorescence intensities >$10^{4}$ in the high FCF (white). (**B**) Illustration of BiFC as a monitoring system for α-Syn oligomerization. BiFC relies on the reconstitution of a Venus fluorophore upon antiparallel dimerization of α-Syn constructs that were fused to non-fluorescent halves of a Venus protein on either the N- or the C-terminus. (**C**) Cell lysis and sample preparation. 48 h after transfection, cells were lysed with RIPA buffer and centrifuged to separate soluble and insoluble (=urea-soluble) protein fractions. The first supernatant was collected as the RIPA-soluble protein fraction. Immunoblots show Venus-tagged α-Syn constructs in the soluble protein fraction of HEK cells expressing BiFC-α-Syn[WT] (upper panel and 1st lane of middle panel). (**C**) After centrifugation, the remaining pellet was washed only once with RIPA buffer as no α-Syn was detected in the supernatants of washing steps 2 and 3. Subsequent addition of UTC buffer REF resolved multimeric α-Syn that was analyzed as the urea-soluble protein fraction (last lanes). Representative ponceau stain shows that also untagged α-Syn was present in both RIPA-soluble and insoluble fractions: multiple washing steps with RIPA buffer could not dissolve all α-Syn species in the lysate as the protein band corresponding to untagged α-Syn (arrow at the right) reappeared after addition of UTC buffer (= urea-soluble fraction in the last lane).

We used Bimolecular Fluorescence Complementation (BiFC) to visualize intracellular α-Syn oligomerization. BiFC relies on α-Syn constructs that are linked to the non-fluorescent halves of the Venus protein. Here, one half is fused to the N-terminus and the corresponding counterpart is fused to the C-terminus of α-Syn. Due to antiparallel α-Syn oligomerization, the formation of an α-Syn dimer reconstitutes a functional Venus fluorophore (Appendix A, Figure A2B and [42]). Accordingly, BiFC is an established method for the direct visualization of α-Syn multimer formation [34,41,44,45]. Since only non-monomeric α-Syn contributes to Venus fluorescence signals, increased fluorescence intensity corresponds to an increase in α-Syn multimerization.

**Figure 1:**
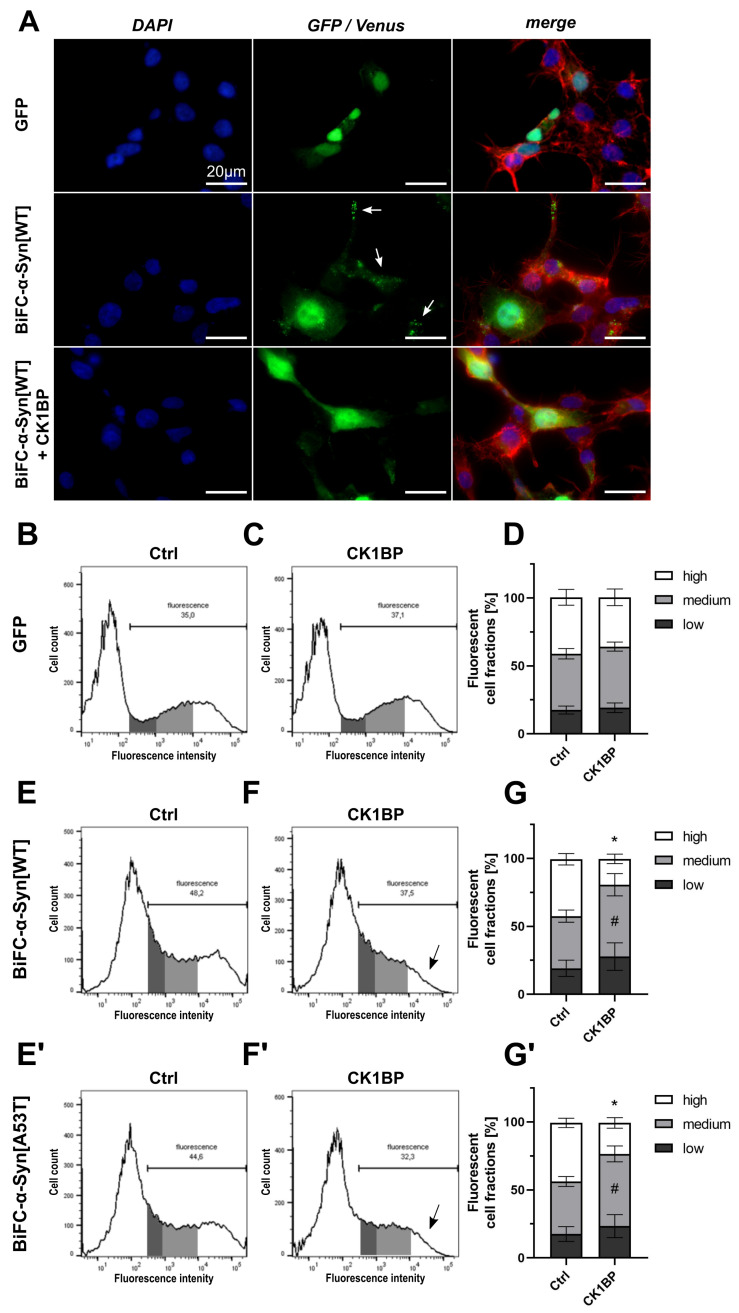
Monitoring intracellular α-Syn oligomerization with Bimolecular Fluorescence Complementation (BiFC). Fluorescence of cells expressing BiFC-α-Syn[WT] and BiFC-α-Syn[A53T] was assessed to measure the effect of CK1 binding protein (CK1BP) on α-Synuclein (α-Syn) oligomerization. (**A**) Epifluorescence microscopy of HEK cells transfected with green fluorescent protein (GFP) (upper panel) or cells co-transfected with BiFC-α-Syn[WT] without (middle panel) or with CK1BP (lower panel). Representative images show nuclear DNA (DAPI, blue), F-actin (Phalloidine, red) and endogenous fluorescence signal (green). Arrows indicate Venus fluorescent foci (“Venus puncta”). Scale bar = 20 µm. (**B**) Representative histogram of GFP-transfected HEK cells demonstrating FACS analysis. Fluorescent cells (right peak) were subdivided into three fluorescent cell fractions (FCFs) with high (white), medium (light grey) or low (dark grey) fluorescence intensity. (**C**) Representative histogram of cells co-transfected with GFP and CK1BP. (**D**) FACS analysis of cells co-transfected with GFP and empty vector control (left bar) or CK1BP (right bar). Bars show FCFs relative to fluorescent cells of the respective condition. FCFs are presented as mean ± SD of six independent experiments with three replicates each. Representative histograms of HEK cells co-transfected with **(E,F)** BiFC-α-Syn[WT] or (**E′**,**F′**) BiFC-α-Syn[A53T] and (**E**,**E′**) empty vector control or (**F**,**F′**) CK1BP. Arrows in (**F**,**F′**) indicate decreased high FCF in CK1BP groups. FACS analysis of cells co-transfected with (**G**) BiFC-α-Syn[WT] or (**G′**) BiFC-α-Syn[A53T] and empty vector control (Ctrl; left bar) or CK1BP (right bar). Bars show FCFs relative to fluorescent cells of the respective condition. FCFs are presented as mean ± SD of five independent experiments with two replicates each. Two-way ANOVA followed by Tukey’s multiple comparisons test was used to determine statistical significance. Significant differences (p<0.05) between FCFs of the respective Ctrl and CK1BP groups are indicated with * comparing high FCFs and # comparing medium FCFs.

Using epifluorescence microscopy, HEK cells transfected with BiFC-α-Syn[WT] in combination with CK1BP or an empty vector control showed fluorescent signals, indicating α-Syn multimerization. Venus fluorescence appeared mainly diffuse in the cytoplasm and nucleus (Figure 1A) as reported previously [41,45]. Additionally, fluorescent cells expressing BiFC-α-Syn[WT] alone regularly displayed intracellular foci with increased Venus fluorescence signal intensities (“Venus puncta”) 48 h after transfection, which could indicate the sites of increased α-Syn accumulation. Co-transfection with CK1BP reduced the frequency of these sites, resulting in a predominantly diffuse green fluorescent signal. This observation suggested that the abundance of α-Syn[WT] multimers was reduced with the overexpression of CK1BP.

BiFC-α-Syn combined with flow cytometry allowed us to analyze Venus signal intensities in an unbiased fashion in large numbers of individual cells. In a representative FACS analysis of cells transfected with GFP, fluorescent cells were separated from non-fluorescent cells (Appendix A, Figure A2A). Previously, BiFC fluorescence has been quantified by determining the mean fluorescence intensity of cells as a measure for protein oligomerization [41,45]. Given the wide range of α-Syn oligomers detected in cells, we chose a two-step approach to analyze BiFC fluorescence. We determined the total percentage of fluorescent cells and then further divided this population into three subgroups according to fluorescence intensity. Low, medium and high fluorescent cell fractions (FCFs) were expressed as proportions relative to all fluorescent cells in a sample to allow a better comparison between conditions (Appendix A, Figure A2A). Thus, this approach permitted us to quantify and compare intracellular α-Syn oligomerization between different conditions.

**Figure A3:**
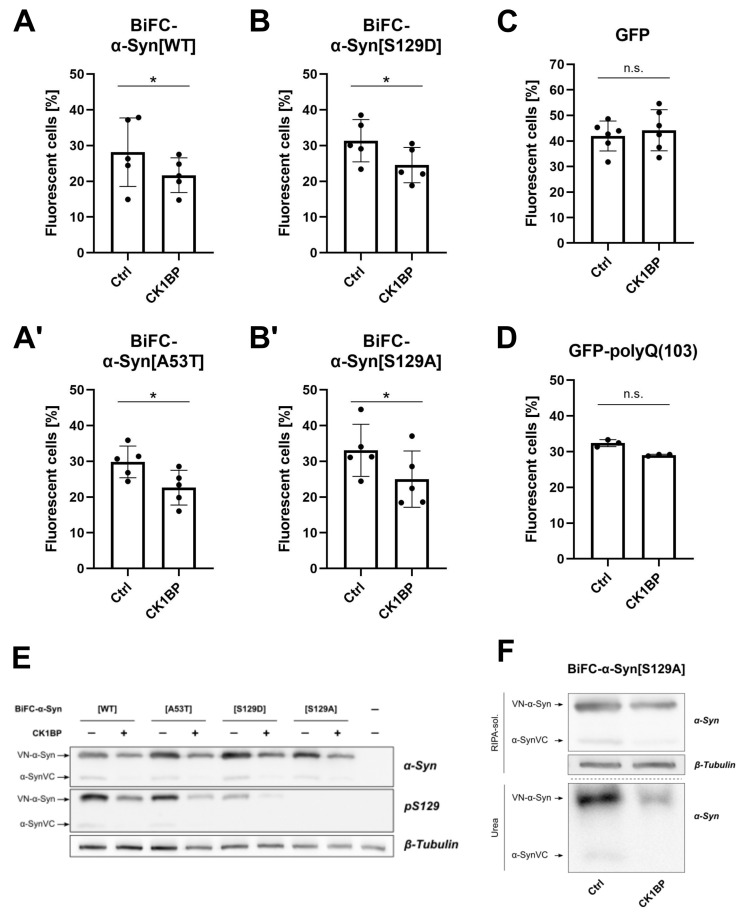
CK1BP decreases abundance of BiFC-α-Syn oligomers. Percentage of fluorescent cells after co-transfection of (**A**) BiFC-α-Syn[WT], (**A′**) BiFC-α-Syn[A53T], (**B**) BiFC-α-Syn[S129D], (**B′**) BiFC-α-Syn[S129A], (**C**) GFP or (**D**) GFP-polyQ and empty vector control (Ctrl) or CK1BP, presented as mean ± SD of at least three independent experiments. One-tailed Wilcoxon matched-pairs signed rank test was used to determine statistical significance of differences between respective Ctrl and CK1BP FACS groups. n.s. not significant, * p<0.05. (**E**) Representative immunoblots displaying protein abundance of total and S129-phosphorylated α-Syn in the RIPA-soluble protein fraction of HEK cells transfected with BiFC-α-Syn and CK1BP (+) or empty vector control (−). Arrows indicate the protein bands that correspond to the respective α-Syn constructs linked to an incomplete Venus protein at the N-(VN-α-Syn) or the C-terminus (α-SynVC). (**F**) Representative immunoblots displaying total soluble and insoluble protein levels of VN-α-Syn and α-SynVC in cells overexpressing BiFC-α-Syn[S129A] with or without CK1BP.

First, we wanted to rule out any non-specific effects of CK1BP on general protein turnover. The concomitant expression of CK1BP and green fluorescent protein (GFP) did not alter the distribution of fluorescent cells with regard to their fluorescence intensities as fluorescent cells formed bell-shaped slopes in the absence (Figure 1B) and presence of overexpressed CK1BP (Figure 1C). This finding suggested that CK1BP did not a ffect general protein turnover. The similar percentage of fluorescent cells in mock and CK1BP co-transfected cells also implied that CK1BP did not affect transfection efficiency in HEK cells (Appendix A, Figure A3C). Moreover, the quantification of cells within the FCFs showed no significant differences comparing cells with and without CK1BP (Figure 1D, high FCFs: p=0.3453).

Next, we analyzed cells expressing BiFC-α-Syn by flow cytometry to monitor the impact of CK1BP on α-Syn oligomerization. In addition to α-Syn[WT], we used the α-Syn[A53T] variant that is associated with familial PD and suspected to have aggregation-enhancing properties [7,19,20,46].

First of all, we realized that the overexpression of CK1BP caused a small but significant reduction in the percentage of fluorescent cells in FACS analysis (Appendix A, Figure A3A,A′). Cells expressing BiFC-α-Syn[WT] in the absence of CK1BP displayed a peaked distribution of fluorescent cells when plotted against fluorescence signal intensity (Figure 1E). The co-expression of CK1BP changed the slope of the curve, as highly fluorescent cells were less abundant (Figure 1F, arrow). The quantification of cells within the different FCFs exposed a significantly decreased proportion of highly fluorescent cells upon CK1BP overexpression compared to the control (Figure 1G, high FCFs: p=0.0001; Figure 1G′, high FCF: p<0.0001). This reduction in high FCF coincided with a significant increase in the relative amount of cells within the medium FCF for both α-Syn variants (Figure 1G, medium FCFs: p=0.0194; Figure 1G′, medium FCFs: p=0.0036). At the same time, the proportion of low-fluorescence cells was not significantly altered by CK1BP overexpression (Figure 1G). Similarly, the co-expression of CK1BP significantly reduced the high FCF of cells expressing α-Syn[A53T] while increasing the proportion of cells with medium fluorescence signal intensities (Figure 1E′–G′). Thus, a shift in cell fractions with high and medium fluorescence signal intensities was observed independent of the BiFC-α-Syn variant tested.

**Figure 2:**
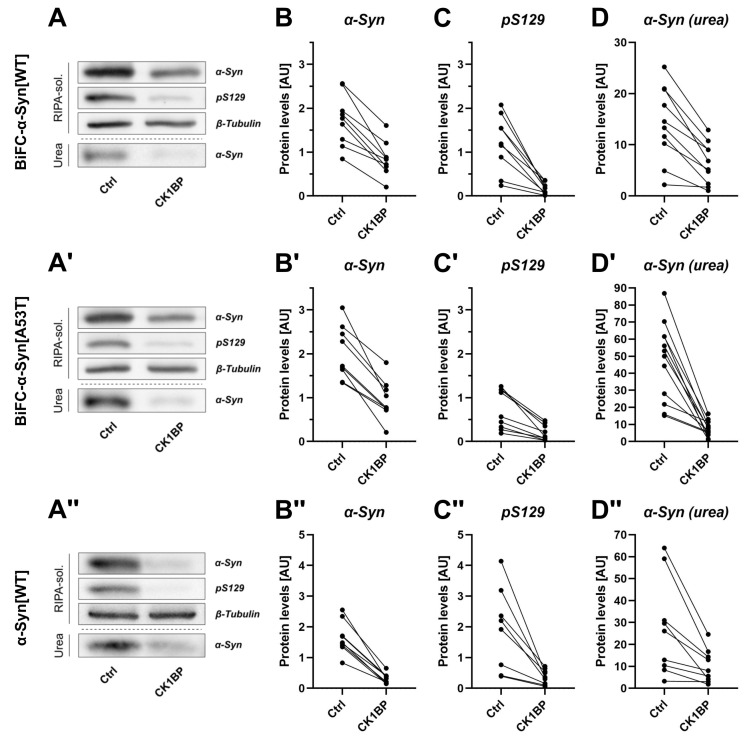
CK1BP decreases S129 phosphorylation and total abundance of α-Syn in the soluble and insoluble protein fraction. Biochemical analysis of differentially fractionized cell lysates was performed to investigate the impact of CK1BP on α-Syn S129 phosphorylation and abundance of detergent-soluble and insoluble α-Syn species. Protein levels of samples co-transfected with CK1BP were compared to α-Syn-only transfected controls (Ctrl). Representative immunoblots displaying protein abundance of α-Syn and S129-phosphorylated α-Syn in the RIPA-soluble and in the urea protein fraction for (**A**) BiFC-α-Syn[WT], (**A′**) BiFC-α-Syn[A53T] and (**A″**) untagged α-Syn[WT]. Quantification of protein levels of soluble (**B**,**B′**,**B″**) α-Syn and (**C**,**C′**,**C″**) S129-phosphorylated α-Syn as well as (**D**,**D′**,**D″**) RIPA-insoluble α-Syn in the urea fraction for (**A**–**D**) BiFC-α-Syn[WT], (**A′**–**D′**) BiFC-α-Syn[A53T] or (**A″**–**D″**) untagged α-Syn[WT]. A total of three to four independent experiments with three technical replicates per experiment were plotted. Outliers in Western Blot band intensity quantification datasets were excluded as identified by ROUT method.

In addition to FACS analysis, we performed SDS-PAGE and subsequent immunoblotting to determine solubility of BiFC-α-Syn (Figure 2A,A′ and Appendix A, Figure A2C). After cell lysis with RIPA buffer, we performed a differential extraction of RIPA-soluble and RIPA-insoluble protein fractions to distinguish detergent-soluble from detergent-insoluble forms of α-Syn via a Western Blot (Appendix A, Figure A2C). The RIPA-soluble protein fraction contained α-Syn species of low to middle molecular weights (MWs) whereas α-Syn oligomers of higher MWs were most likely present in the RIPA-insoluble lysate fraction that was dissolved in UTC buffer [10,23]. The quantitative analysis showed that CK1BP overexpression considerably reduced the total amount of soluble α-Syn in comparison to the control (>50% reduction; Figure 2B,B′). When we analyzed the soluble protein levels in both conditions, we observed a strong decrease in the abundance of S129-phosphorylated α-Syn (>70% reduction; Figure 2C,C′) induced by CK1BP overexpression. The protein levels of RIPA-insoluble α-Syn were also reduced (>50% reduction) for both BiFC-α-Syn[WT] and BiFC-α-Syn[A53T] (Figure 2D, D′) in the case of CK1BP co-expression. In summary, the overexpression of CK1BP strongly reduced the amount of BiFC-α-Syn in the RIPA-soluble as well as in the RIPA-insoluble fraction. This effect was independent of the α-Syn variant (WT and A53T) used.

To reject the proposal that the Venus tags might have contributed to the observed CK1BP-mediated decrease in BiFC-α-Syn levels, we performed the same experiments using untagged α-Syn[WT] (Figure 2A″–D″). Similar to the effects observed with BiFC-α-Syn, we found that co-expression of the untagged construct with CK1BP caused a significant reduction in α-Syn abundance compared to the control. This reduction was observed for total α-Syn (Figure 2B″) and S129-phosphorylated α-Syn (Figure 2C″) in the soluble fraction as well as for RIPA-insoluble α-Syn (Figure 2D″). This finding suggested that the reduction in RIPA-soluble and RIPA-insoluble protein levels of α-Syn in CK1BP-overexpressing cells was unaffected by the Venus tags. Taken together, these results supported the hypothesis that the CK1BP-mediated inhibition of CK1 kinase activity decreased α-Syn S129 phosphorylation as well as the abundance of non-monomeric α-Syn.

### 3.2. CK1BP Reduces α-Syn Accumulation Independent of S129 Phosphorylation

To further investigate the relevance of S129 phosphorylation for α-Syn oligomerization, we analyzed the effect of CK1BP on BiFC-α-Syn with S129 mutations. We used a phosphomimetic α-Syn variant with serine 129 mutated to aspartic acid (BiFC-α-Syn[S129D]) to mimic the constitutive phosphorylation of amino acid 129. In addition, we used α-Syn[S129A], where serine is replaced by alanine to abolish phosphorylation at position 129. We chose both variants to determine whether the observed CK1BP-mediated decrease in α-Syn levels depended on the presence of a phospho(mimetic) site of α-Syn at amino acid 129.

Biochemical analyses confirmed the specificity of our pS129 antibody. In the Western Blot analysis, the pS129-specific antibody detected α-Syn[S129D] (although with reduced efficacy compared to a genuine S129 phosphorylation of α-Syn) and obviously failed to detect α-Syn[S129A] (Appendix A, Figure A3E).

**Figure 3:**
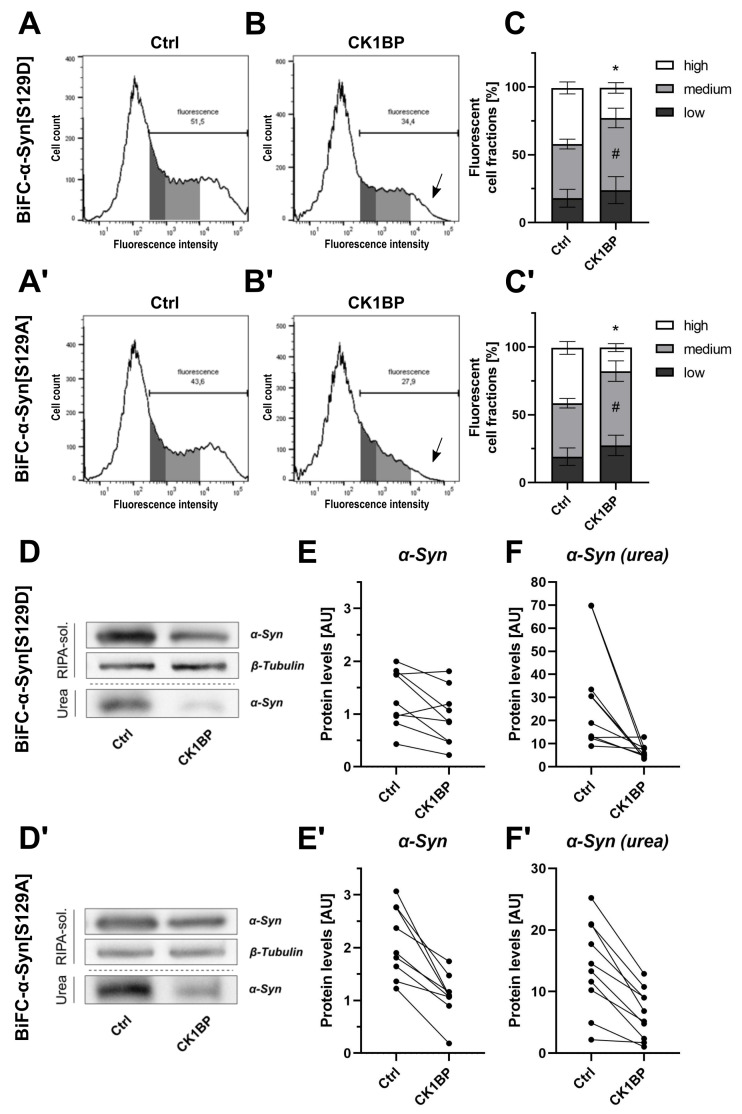
CK1BP reduces α-Syn accumulation independent of S129 phosphorylation. Cells expressing BiFC-α-Syn[S129D] or BiFC-α-Syn[S129A] were analyzed to determine the relevance of α-Syn S129 phosphorylation for the CK1BP-related decrease in α-Syn accumulation. Representative histograms of HEK cells co-transfected with (**A**,**B**) BiFC-α-Syn[S129D] or (**A′**,**B′**) α-Syn[S129A] and (**A**,**A′**) empty vector control or (**B**,**B′**) CK1BP. Arrows in (**B**,**B′**) indicate decreased high FCF in CK1BP groups. FACS analysis of cells co-transfected with (**C**) BiFC-α-Syn[S129D] or (**C′**) BiFC-α-Syn[S129A] and empty vector control (left bar) or CK1BP (right bar). Bars show FCFs relative to fluorescent cells of the respective condition. FCFs are presented as mean ± SD of five independent experiments with two replicates each. Two-way ANOVA followed by Tukey’s multiple comparisons test was used to determine statistical significance. Significant differences (p<0.05) between FCFs of the respective Ctrl and CK1BP groups are indicated with * comparing high FCFs and # comparing medium FCFs. Representative immunoblots displaying protein abundance of (**D**) BiFC-α-Syn[S129D] or (**D′**) BiFC-α-Syn[S129A] in the RIPA-soluble and in the urea protein fraction. Quantification of protein levels of (**E**,**E′**) soluble α-Syn and (**F**,**F′**) RIPA-insoluble α-Syn in the urea fraction of cells expressing (**D**–**F**) BiFC-α-Syn[S129D] or (**D′**–**F′**) BiFC-α-Syn[S129A]. A total of three (**E**,**E′**) or four (**F**,**F′**) independent experiments with three technical replicates per experiment were plotted. Outliers in Western Blot band intensity quantification datasets were excluded as identified by ROUT method.

We used FACS analysis to determine the fluorescence intensities of cells expressing these BiFC-α-Syn variants with (Figure 3A,A′) or without (Figure 3B,B′) the concomitant expression of CK1BP. Interestingly, CK1BP altered the fluorescence signals of cells expressing BiFC-α-Syn[S129D] as well as BiFC-α-Syn[S129A]. The co-transfection of CK1BP slightly, but significantly, reduced the amount of fluorescent cells for both α-Syn phosphomutants (Appendix A, Figure A3B,B′). The same effect had been observed with aSyn[WT] and aSyn[A53T] (Appendix A, Figure A3A,A′), but not in cells expressing non-aggregating GFP (Appendix A, Figure A3C). Moreover, CK1BP significantly reduced the proportion of cells with high fluorescence intensities in cells overexpressing α-Syn[S129D] or α-Syn[S129A] compared to control conditions (Figure 3C, high FCFs: p=0.0009; Figure 3C′, high FCFs: p<0.0001). Similar to α-Syn[WT] and α-Syn[A53T], this decrease in high FCF went along with a relative increase in the amount of cells within the medium FCF for both α-Syn[S129D] (Figure 3C, medium FCFs: p=0.0315) and BiFC-α-Syn[S129A] (Figure 3C′, medium FCFs: p=0.0037).

The corresponding biochemical analyses demonstrated that CK1BP reduced α-Syn abundance in both the RIPA-soluble and urea protein fractions of cells expressing either BiFC-α-Syn[S129D] (Figure 3D–F) or BiFC-α-Syn[S129A] (Figure 3D′–F′).

In experiments using BiFC-α-Syn, the characterization of the respective fluorescent cell populations by FACS analysis allowed a clear distinction between control conditions and samples co-transfected with BiFC-α-Syn and CK1BP. The differences observed were similar for all α-Syn variants studied (about 50% reduction in high FCF). Moreover, the biochemical analyses of RIPA-soluble and insoluble α-Syn implied similar protein levels of overexpressed α-Syn and a comparable reduction in multimeric α-Syn upon CK1BP co-expression for α-Syn[WT] and α-Syn[A53T] as well as α-Syn[S129D] and α-Syn[S129A] (Appendix A, Figure A3E,F).

We initially hypothesized that, by inhibiting CK1 kinase activity, CK1BP would reduce the S129 phosphorylation of α-Syn and thereby might affect intracellular α-Syn accumulation (scheme in Appendix A, Figure A1A). Indeed, the abundance of S129-phosphorylated α-Syn was reduced in cells co-expressing CK1BP and α-Syn[WT] or α-Syn[A53T]. In parallel, we observed a reduction in highly fluorescent cells and a decrease in insoluble a-Syn levels when overexpressing CK1BP (Figure 1 and Figure 2). However, we obtained similar results when co-expressing CK1BP with α-Syn[S129D] or α-Syn[S129A] (Figure 3). Here, we could exclude changes in the phosphorylation status of α-Syn at position 129. Nevertheless, the overexpression of CK1BP caused a reduction in multimeric α-Syn in FACS analysis and a decrease in insoluble (aggregated) α-Syn. These findings suggested that the CK1BP-mediated effects on α-Syn accumulation might be independent of the S129 phosphorylation of α-Syn.

### 3.3. CK1BP Decreases Accumulation of a Polyglutamine Peptide

To further elucidate the role of CK1BP in intracellular protein accumulation, we extended our analysis to another aggregating protein. We chose a polyglutamine (polyQ) peptide as it lacked a Ser/Thr phosphorylation site. A common feature of polyglutamine diseases is CAG repeat expansions within the coding regions of the respective disease-linked genes. As CAG codes for glutamine, elongatd polyQ stretches are the main characteristic of disease-associated proteins. The lengths of these polyQ stretches in the respective disease-associated proteins determine clinical manifestation and correlate with disease onset and severity. Thus, elongated polyQ stretches and subsequent protein misfolding are the common pathological hallmarks of polyglutamine disease, an otherwise heterogenic group of NDDs [47].

**Figure 4:**
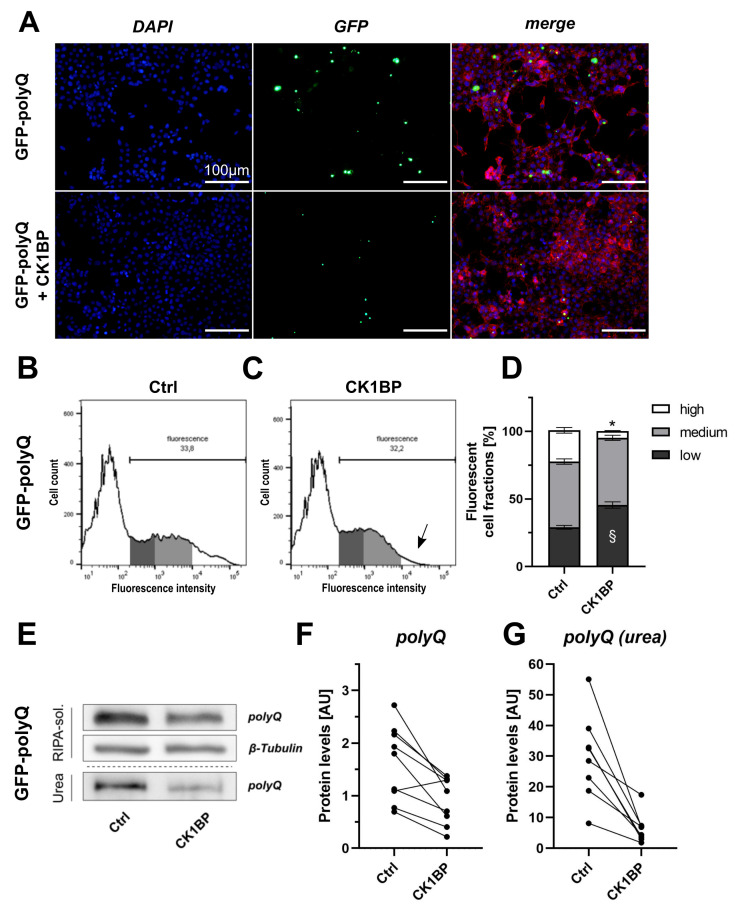
CK1BP decreases accumulation of a polyglutamine (polyQ) peptide. Fluorescence and biochemical analysis were used to investigate the impact of CK1BP on intracellular abundance of an aggregating GFP-polyQ fusion protein. (**A**) Epifluorescence microscopy of HEK cells co-transfected with GFP-polyQ and empty vector control (upper panel) or CK1BP (lower panel). Representative images show nuclear DNA (DAPI, blue), F-actin (Phalloidine, red) and endogenous fluorescence signal (green). Scale bar = 100 µm. Representative histograms of HEK cells co-transfected with GFP-polyQ and (**B**) empty vector control or (**C**) CK1BP. Arrow in (**C**) indicates decreased high FCF in CK1BP group. (**D**) FACS analysis of cells co-transfected with GFP-polyQ and empty vector control (left bar) or CK1BP (right bar). Bars show FCFs relative to fluorescent cells of the respective condition. FCFs are presented as mean ± SD of three independent experiments with three replicates each. Two-way ANOVA followed by Tukey’s multiple comparisons test was used to determine statistical significance. Significant differences (p<0.05) between FCFs of the respective Ctrl and CK1BP groups are indicated with * comparing high FCFs and § comparing low FCFs. (**E**) Representative immunoblots displaying protein abundance of GFP-polyQ in the RIPA-soluble and in the urea protein fraction. Quantification of protein levels of (**F**) soluble polyQ and (**G**) insoluble polyQ in the urea fraction. A total of three independent experiments with three technical replicates per experiment were plotted. Outliers in Western Blot band intensity quantification datasets were excluded as identified by ROUT method.

We used a Huntingtin exon 1-derived peptide with a polyQ stretch of 103 repeats fused to a green fluorescent protein (GFP) (GFP-polyQ). This construct was used to analyze the effect of CK1BP on polyQ protein aggregation. HEK cells expressing GFP-polyQ presented with large, highly fluorescent intracellular inclusions 48 h after transfection (Figure 4A). This accumulation was triggered by the polyQ peptide as cells transfected with GFP alone displayed an evenly distributed green fluorescence signal and lacked highly fluorescent protein accumulations (Figure 1A). The co-expression of CK1BP and GFP-polyQ decreased the number of cells displaying fluorescent inclusions and considerably reduced the sizes of these fluorescent foci (exemplarily shown in Figure 4A).

As for BiFC-α-Syn variants, we quantified fluorescence signal intensities by FACS analysis (Figure 4B,C). The co-expression of CK1BP significantly reduced the proportion of highly fluorescent cells (>80% reduction) compared to cells expressing GFP-polyQ alone (Figure 4D, high FCFs: p<0.0001). This mirrored the trend observed in our previous analyses using BiFC-α-Syn variants. However, there were differences in the distribution of cells in the other FCFs. In the case of BiFC-α-Syn, the CK1BP-mediated decrease in the high FCF went along with an augment in the medium FCF. In cells expressing GFP-polyQ on the other hand, the CK1BP-mediated reduction in cells within the high FCF was accompanied by a relative increase in the amount of low-fluorescence cells (Figure 4D, low FCFs: p<0.0001, medium FCFs: p=0.9761).

Additionally, the overexpression of CK1BP significantly decreased the relative amount of fluorescent cells when using BiFC-α-Syn variants (Appendix A, Figure A3A–B′). In contrast, the co-expression of CK1BP with GFP-polyQ did not reduce the percentage of fluorescent cells compared to the control (Appendix A, Figure A3D). This is in line with our previous observations when analyzing GFP expressing cells with or without the co-expression of CK1BP (Appendix A, Figure A3C). Note that, unlike BiFC-α-Syn, monomeric GFP-polyQ also contributed to the fluorescence signals obtained by flow cytometry. Thus, FACS analysis suggested that CK1BP did not seem to affect the monomeric species of the aggregating GFP-polyQ protein. We therefore hypothesized that the regulation of protein expression was not a major mechanism responsible for the reduced levels of aggregation-prone proteins seen with CK1BP overexpression. Conversely, CK1BP seemed to specifically affect protein aggregation as CK1BP overexpression did not alter the fluorescence intensity of cells transfected with GFP.

The biochemical analysis of GFP-polyQ protein levels (Figure 4E) in fractionized cell lysates indicated a reduced abundance of RIPA-soluble polyQ (>40%, Figure 4F) upon CK1BP co-transfection. In addition, CK1BP co-transfection strongly reduced polyQ protein levels in the RIPA-insoluble fraction (>75%, Figure 4G) compared to the control.

In summary, our analyses showed that CK1BP reduced the oligomerization of α-Syn[WT] and α-Syn[A53T]. The observed reduction in multimeric α-Syn levels was independent of S129 phosphorylation as similar results were obtained with S129 phosphomutants α-Syn[S129D] and α-Syn[S129A]. Moreover, CK1BP also reduced the aggregation of a polyQ peptide. Taken together, our results suggest that CK1BP overexpression affected the intracellular accumulation of aggregation-prone proteins that are of pathological relevance in NDDs.

## 4. Discussion

Conformational changes in soluble α-Syn are thought to initiate α-Syn aggregation by enabling the oligomerization and eventual deposition of α-Syn species in insoluble aggregates [15,19]. In vitro data on and observations of living cells suggest a highly dynamic process of oligomer formation of varying sizes and morphologies that progress into larger aggregates. At the same time, the accumulation process seems to be reversible and already formed α-Syn multimers can disintegrate into smaller species [14,16]. In view of the dynamics and complexity of the α-Syn aggregation process, we used BiFC to assess the oligomerization of different α-Syn mutations in vivo. We evaluated the effect of CK1BP overexpression on α-Syn multimerization by semiquantitative FACS analysis and biochemical analyses of fractionized cell lysates.

BiFC was originally developed as a screening system to identify protein–protein interactions [48]. In 2008, Outeiro et al. were the first to use BiFC for the monitoring of intracellular α-Syn oligomerization [41]. Since then, the technique has been continuously modified and improved to study α-Syn accumulation in various models [34,44,45]. Recently, it has been questioned whether BiFC is a fitting tool to model disease-relevant α-Syn aggregation [42]. BiFC signals in cell culture experiments were mostly homogenous and it is unclear to what extent BiFC oligomers proceed into disease-like α-Syn aggregates [41,45,49]. Instead, inclusion formation was often achieved by applying additional methods to BiFC systems [45]. However, punctate fluorescence signals and aggregated α-Syn were observed in BiFC animal models [34,44,50,51]. By using the epifluorescence microscopy of cells transfected with BiFC-α-Syn[WT], we detected fluorescent foci that were not present in cells expressing soluble GFP (Figure 1A). In view of the time-dependent multimerization of α-Syn, the Venus puncta detected in our system 48 h after transfection most likely represented higher-ordered oligomers of BiFC-α-Syn [16,19,34]. Although fluorescence microscopy allows us to visualize fluorescent patterns in individual cells, the quantification of fluorescent cells from microscopical images meets certain challenges. First, the correct allocation of fluorescence signals to single cells relies on optimal cell distribution within the culture area. Second, the manual categorization of cells according to criteria such as cell fluorescence and the presence of a certain number or size of fluorescent foci per cell depends on stable fluorescent signals and is prone to observation bias.

FACS analysis, however, represents an objective method for fluorescence detection in high cell numbers. While also detecting fluorescence signals from individual cells, automatization ensures minimal bleaching of fluorescent complexes in the measurement process. Therefore, we used FACS analysis to investigate how CK1BP might affect multimerization and the corresponding fluorescence signal of cells expressing BiFC-α-Syn. Moreover, FACS analysis allows one to generate a fluorescence profile of a cell population by enabling the precise detection of single-cell fluorescence intensities. For better comparison between different conditions and experiments, we grouped fluorescent cells into three fluorescent cell fractions (Appendix A, Figure A2A). This allowed us to assess potential changes in fluorescence intensities independent of transfection efficiencies. At the same time, the FACS analysis of BiFC-expressing cells as performed here did not provide information on the specific nature of the multimeric α-Syn species. However, an increased amount of at least dimerized α-Syn will increase the fluorescence intensity of an individual cell. Accordingly, in our FACS analysis of BiFC experiments, the highly fluorescent cell fraction (FCF) represented cells with elevated levels of non-monomeric α-Syn. Based on the stated conversion of small oligomeric α-Syn species into larger accumulations, we speculated that higher ordered forms of α-Syn would also be enriched in these cells compared to cells with lower fluorescence intensities. In cells expressing BiFC-α-Syn[WT], the overexpression of CK1BP caused a relative reduction in highly fluorescent cells (Figure 1E′,F′). This decrease might reflect either a simple reduction in the amount of α-Syn multimers per cell or a concomitant reduction in the size of intracellular α-Syn oligomers.

To assess this question, we used a different fluorescence-based reporter system and analyzed a polyQ fusion protein (Figure 4). As observed with BiFC-α-Syn, there was a decrease in the high FCF of cells expressing GFP-polyQ after CK1BP co-transfection (Figure 4B–D). The microscopical images of the respective conditions suggested that CK1BP reduced the abundance and fluorescence intensity of GFP-polyQ accumulations (Figure 4A). These analyses on GFP-polyQ imply that CK1BP most likely decreased both the abundance and size of intracellular α-Syn oligomers. This conclusion was consolidated by the decrease in protein levels that we observed for both soluble and insoluble BiFC-α-Syn and GFP-polyQ (Figure 2, Figure 3 and Figure 4). Thus, the immunoblotting of fractionized cell lysates revealed the effect of CK1BP overexpression on α-Syn oligomers of different solubilities, comprising potentially pathogenic species [20,21,22]. However, the sample preparation performed here prevented the size differentiation of α-Syn species in the respective protein fractions as well as the individual detection of monomeric or dimeric α-Syn [43]. Nevertheless, we observed a significant reduction in the relative amount of fluorescent cells in CK1BP/BiFC-α-Syn co-expressing conditions (Appendix A, Figure A3A–B′). In our BiFC system, fluorescence signals are only detected upon α-Syn dimerization or the formation of higher ordered α-Syn multimers, whereas GFP is fluorescent as a monomer. Accordingly, CK1BP did not affect transfection efficiency as cells expressing GFP or GFP-polyQ showed a stable proportion of fluorescent cells regardless of co-transfection with CK1BP (Appendix A, Figure A3C,D). Furthermore, CK1BP did not alter the distribution of fluorescent cells when co-expressed with GFP alone (Figure 1B–D). These findings suggest that CK1BP specifically affects aggregated proteins. In particular, CK1BP appears to promote α-Syn degradation or impede α-Syn oligomerization at a stage as early as dimer formation.

Another issue of BiFC systems is the fact that the Venus tags could alter the stability of α-Syn and fluorophore complementation might be at least partially irreversible [41,48,52]. While the extended half-life of BiFC constructs facilitates the detection of weak protein–protein interactions, it might also stabilize protein oligomers. In our study, protein levels of VN-α-Syn were more stable and readily detected than α-SynVC (Appendix A, Figure A3E,F), a frequently reported imbalance that cannot be compensated by the plasmid-specific adjustment of protein expression [42]. Likewise, concerns have arisen regarding a suspected tendency of VN constructs to self-oligomerize and the risk of VC monomer entrapment in VN accumulations [42,53]. This might produce fluorescence signals that are not directly attributable to the formation of α-Syn dimers. Although not stoichiometric in nature, such BiFC signals reflect α-Syn accumulation and thus can be compared between conditions with the same baseline. Moreover, VN-α-Syn/α-SynVC dimers were detected in brain lysates of BiFC-α-Syn transgenic (tg) mice and corresponding Venus signals strongly colocalized with oligomeric species and S129-phosphorylated α-Syn [44]. Furthermore, the BiFC-α-Syn tg mice displayed critical genetic alterations characteristic of a PD phenotype [44]. Thus, this BiFC-α-Syn tg mouse model succeeded in reproducing key features of α-Syn pathology. In our study, α-SynVC protein levels—if detected—were affected by CK1BP overexpression to the same extent as VN-α-Syn constructs (shown exemplarily for BiFC-α-Syn[S129A] in Appendix A, Figure A3F). Moreover, the decrease in soluble and insoluble species of BiFC-α-Syn paralleled the changes in protein levels of untagged α-Syn[WT] observed with CK1BP co-expression (Figure 2). Regarding the CK1BP-mediated decrease in α-Syn accumulation, we therefore conclude that the outcome of our experiments was not affected by the aforementioned distinct features of BiFC-α-Syn and VN-α-Syn.

Previous research suggested that familial PD α-Syn[A53T] mutation might alter α-Syn aggregation, thereby potentially contributing to increased in vivo neurotoxicity [19,20,46]. In our model, the overexpression of CK1BP reduced the oligomerization and abundance of both α-Syn[A53T] and α-Syn[WT]. In particular, there was a marked CK1BP-induced decrease in α-Syn[A53T] levels in the insoluble protein fraction (Figure 2D′), similar to the trend observed with α-Syn[WT] (Figure 2D,D″). Furthermore, both BiFC-α-Syn variants showed a clear reduction in α-Syn multimers upon CK1BP overexpression, as reflected by the shifts in fluorescence intensities (Figure 1, Appendix A, Figure A3A,A′). Nonetheless, our study did not provide insight into the specific oligomerization process as we only compared α-Syn protein level and solubility at a specific time point. Although our findings suggest otherwise, we therefore cannot fully exclude potential differences between α-Syn[WT] and α-Syn[A53T] in the way that CK1BP affects oligomer formation.

One of our objectives was to determine whether CK1BP affected the accumulation of α-Syn by inhibiting S129 phosphorylation via CK1. Although the S129 phosphorylation of α-Syn has been the subject of extensive research, the function of this modification and its relevance for α-Syn pathology remain elusive. Moreover, the phosphorylation of Y125 in α-Syn has been identified as a priming event that is required for S129 phosphorylation by CK1 [54]. Accordingly, S129 phosphorylation appears to be the last step of α-Syn modifications, which are suspected to alter α-Syn properties. Therefore, we decided to concentrate on S129 phosphorylation. We found that the overexpression of CK1BP reduced S129 phosphorylation as well as the total abundance of both α-Syn[WT] and α-Syn[A53T] (Figure 2). In addition, CK1BP has also been reported to inhibit the phosphorylation of α-Syn as well as tau by CK1 in vitro [36]. These observations led us to hypothesize that the inhibition of CK1 kinase activity and a subsequent decrease in S129 phosphorylation might mediate the reduction in α-Syn protein levels induced by the overexpression of CK1BP (Appendix A, Figure A1). To test this hypothesis, we used α-Syn phosphovariants that either mimic or abolish phosphorylation of α-Syn at S129. Phosphomimetics such as α-Syn[S129D] provide a valuable tool to study the effects of S129 phosphorylation and show structural homology to natively S129-phosphorylated α-Syn [25]. Still, they fail to reproduce the exact conformational changes and behavior of the protein that is induced by genuine S129 phosphorylation [25]. The phospho-dead mutant α-Syn[S129A] might help to identify cellular processes that are linked to pathological forms of α-Syn and depend on phosphorylation at S129. However, these phosphomutants cannot replicate the dynamic process of in vivo phosphorylation and dephosphorylation. We analyzed cells expressing α-Syn[S129D] or α-Syn[S129A] with or without CK1BP (Figure 3). Interestingly, we observed a marked CK1BP-induced decrease in oligomerization of the S129 variants as reflected by a reduced proportion of highly fluorescent cells (Figure 3C,C′). Moreover, biochemical analyses revealed that CK1BP reduced the amount of soluble and insoluble α-Syn species of both α-Syn[S129D] (Figure 3D–F) and α-Syn[S129A] (Figure 3D′–F′). If S129 phosphorylation mediated the effect of CK1BP on α-Syn aggregation, the latter should not have been altered by CK1BP overexpression when using S129 phosphomutants instead of α-Syn[WT]. Nevertheless, CK1BP reduced α-Syn accumulation for all the α-Syn variants analyzed (Figure 1, Figure 2 and Figure 3, Appendix A, Figure A3E).

In summary, we show that the overexpression of CK1BP impacted on S129 phosphorylation of α-Syn and also affected its solubility. Our findings are in line with the available evidence suggesting that CK1BP might act via the inhibition of CK1 kinase activity [36]. The overexpression of CK1BP induced a marked reduction in multimeric and insoluble α-Syn species. At the same time, we have recently shown that the co-expression of the fly orthologue of CK1 (*Dco*) and human α-Syn enhances α-Syn aggregation and toxicity in flies [34]. Thus, our data support a role of CK1 in intracellular α-Syn accumulation. Nevertheless, CK1BP also affected S129 mutants of α-Syn. Therefore, our data indicate that the CK1BP effect does not depend on α-Syn phosphorylation at S129.

Albeit our setting allowed us to compare the control conditions of α-Syn[WT] and S129 variants. All BiFC-α-Syn constructs displayed equal expression levels at 48 h after transient transfection (Appendix A, Figure A3E). Furthermore, the FACS analysis displayed a comparable percentage of fluorescent cells for all BiFC-α-Syn variants (Appendix A, Figure A3A–B′) which were equally distributed with regard to fluorescence intensity (Figure 1B,B′, Figure 2A,A′). Thus, our findings suggest that S129 mutations do not affect α-Syn accumulation. These observations are in line with previous findings concluding that S129 phosphorylation might be a secondary event and serve as a biomarker for disease stage rather than playing a causative role in α-Syn aggregation [27,55]. Oueslati and co-workers recently identified S129 phosphorylation as a key modification that enabled PLK2–α-Syn interaction and subsequent co-degradation via the macroautophagy pathway [30]. Moreover, α-Syn ubiquitination partially overlapped with pS129 positivity in soluble and LB-associated α-Syn fractions of disease cases [10,23]. These findings exemplify the numerous possibilities of how S129 phosphorylation could influence α-Syn aggregation. In particular, the cellular processes involved could specifically affect α-Syn homeostasis depending on S129 phosphorylation. In our model, however, such a mechanism is unlikely as CK1BP reduced the accumulation of α-Syn regardless of S129 phosphorylation.

The results from α-Syn experiments prompted us to ask whether the effect of CK1BP was specific for α-Syn. In polyQ diseases, neurodegeneration is associated with the pathological forms of proteins that contain elongated stretches of polyQ [47]. Accordingly, inclusions of polyQ-expanded Huntingtin are associated with Huntington’s disease [47,56]. We examined accumulation of a polyQ(103)-Huntingtin exon 1 fragment fused to GFP. We found that CK1BP reduced the amount of soluble species as well as higher-ordered accumulations of the GFP-polyQ protein (Figure 4). Even though the pathophysiology of α-synucleinopathies and polyglutamine diseases differ in many ways, oligomeric forms of amyloidogenic proteins seem to share common properties and an oligomer-specific pathomechanism has been proposed to explain the detrimental effects of these species on cells [57,58]. As CK1BP modulated the accumulation of both α-syn and a polyQ protein, it could be involved in the regulation of these pathways. In summary, our findings imply that CK1BP is likely to impede accumulation or enhance protein clearance while specifically affecting aggregating proteins.

Thus, our findings make CK1BP an interesting target to study regarding the regulatory mechanisms that influence the intracellular accumulation of disease-associated proteins in NDDs. CK1BP reduced the amount of small soluble as well as higher-ordered α-Syn and polyQ oligomers. The available evidence suggests that CK1BP most likely acts via the inhibition of CK1. Nevertheless, our data do not exclude that the observed reduction in S129 phosphorylation might rather be an indirect effect resulting from the interaction of CK1BP with other kinases that target, e.g., the Y125 of α-Syn [54]. Furthermore, the CK1BP effect on protein aggregation was not specific for α-Syn (Figure 4). Given that our results do not support a direct and specific phosphorylation of the accumulating proteins as the underlying mechanism, further studies are needed to clarify the specifications of this pathway. Unfortunately, to the best of our knowledge, there is no siRNA or inhibitor available that would target all six human CK1 isoforms. Similarly, as CK1 is a highly conserved kinase, there are no CK1-deficient cell lines that are readily available [31]. Such lines could be generated, e.g., by genome editing via CRISPR/CAS9. They might be a valuable tool for further investigation of the cellular functions of CK1 and its interplay with CK1BP. Accordingly, the clarification of the physiological function and the interaction of CK1BP and CK1 as well as their role in neurodegenerative diseases are interesting subjects for further research.

We showed that, especially when combined with other fluorescence-based and biochemical methods, the FACS analysis of HEK cells overexpressing BiFC-α-Syn allows one to study early steps in α-Syn oligomerization that might be pathogenically relevant [19,59]. This method could be refined for future research. For example, flow cytometry allows the determination of cellular granularity by measuring the side scatter of a cell. Thus, in combination with fluorescent systems such as BiFC, FACS analysis provides an opportunity to further characterize intracellular protein accumulation. Nevertheless, there are some limitations to the BiFC model and other approaches will be needed to determine the impact of CK1BP on α-Syn (neuro)toxicity and the formation of α-Syn aggregates beyond the oligomeric species described herein.

In view of the scarce knowledge on interaction partners of CK1BP [60] and its physiological function, further investigation of the CK1BP interactome is another objective for future research. Eventually, the characterization of CK1BP in the human CNS may contribute to a better understanding of neurodegenerative processes and might lead to new therapeutic approaches.

## Data Availability

The data presented in this study are available on request from the corresponding author.

## Supplementary Materials

The following are available online at https://www.mdpi.com/article/10.3390/cells10112830/s1, Supplementary data file: Original Western Blot membrane images.
