# Supplementary material for: CK1BP Reduces α-Synuclein Oligomerization and Aggregation Independent of Serine 129 Phosphorylation
Source: Cells. 2021 Oct 21;10(11):2830. doi: 10.3390/cells10112830 (PMC8616157; doi:10.3390/cells10112830)

Figure 2A (BiFC-α-Syn[WT])

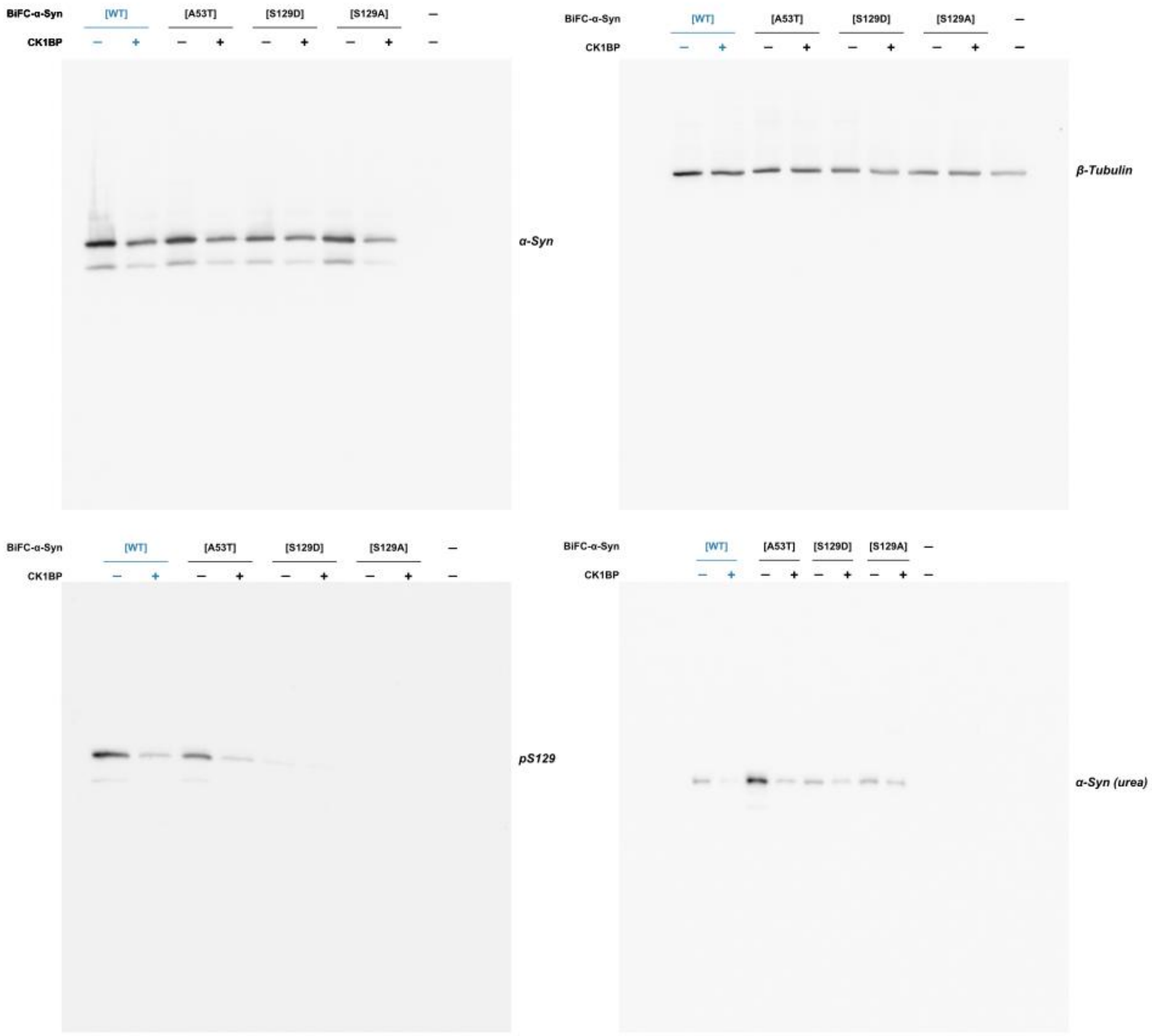

Figure 2A' (BiFC-α-Syn[A53T])

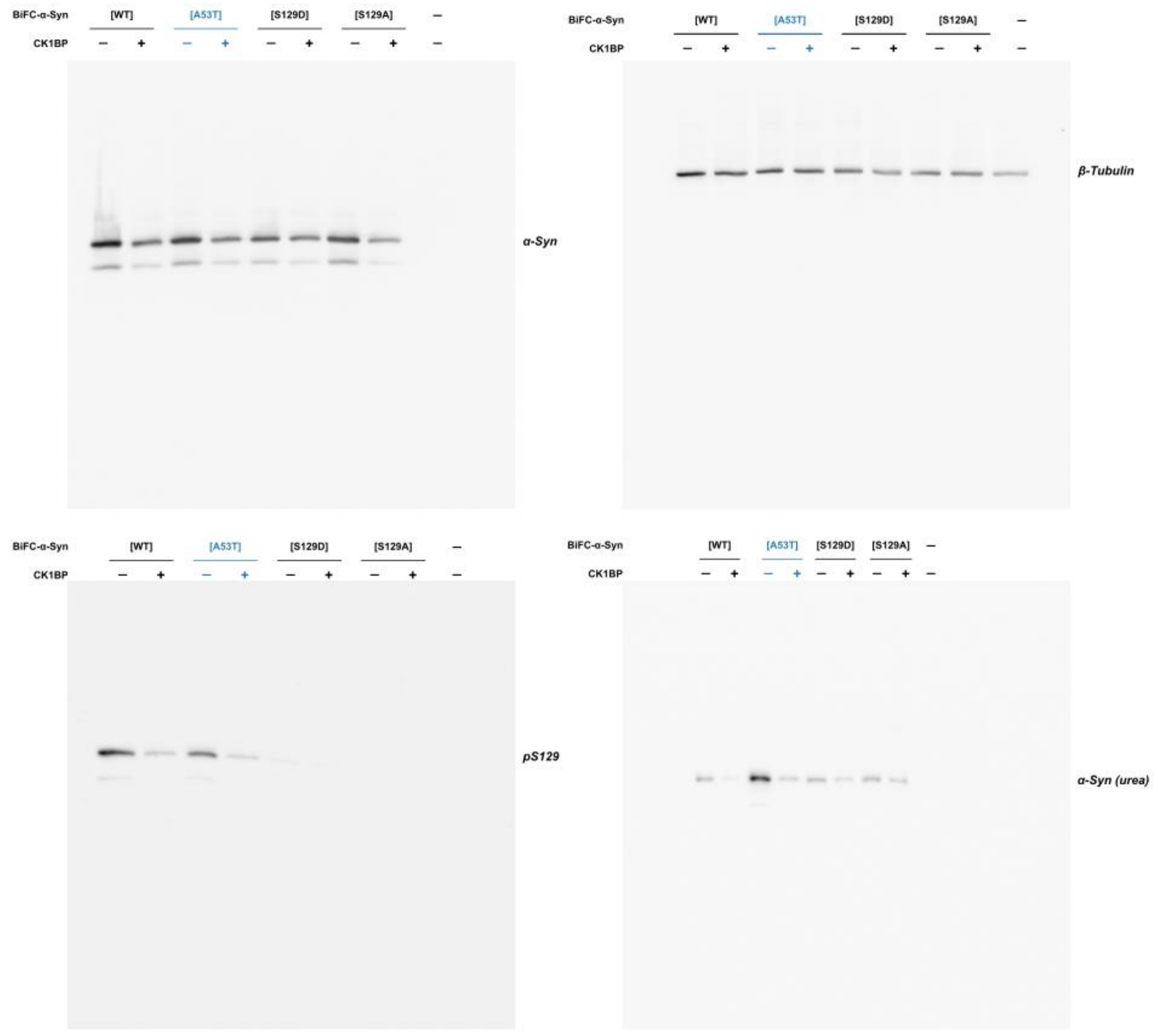

Figure 2A'' (untagged α-Syn[WT])

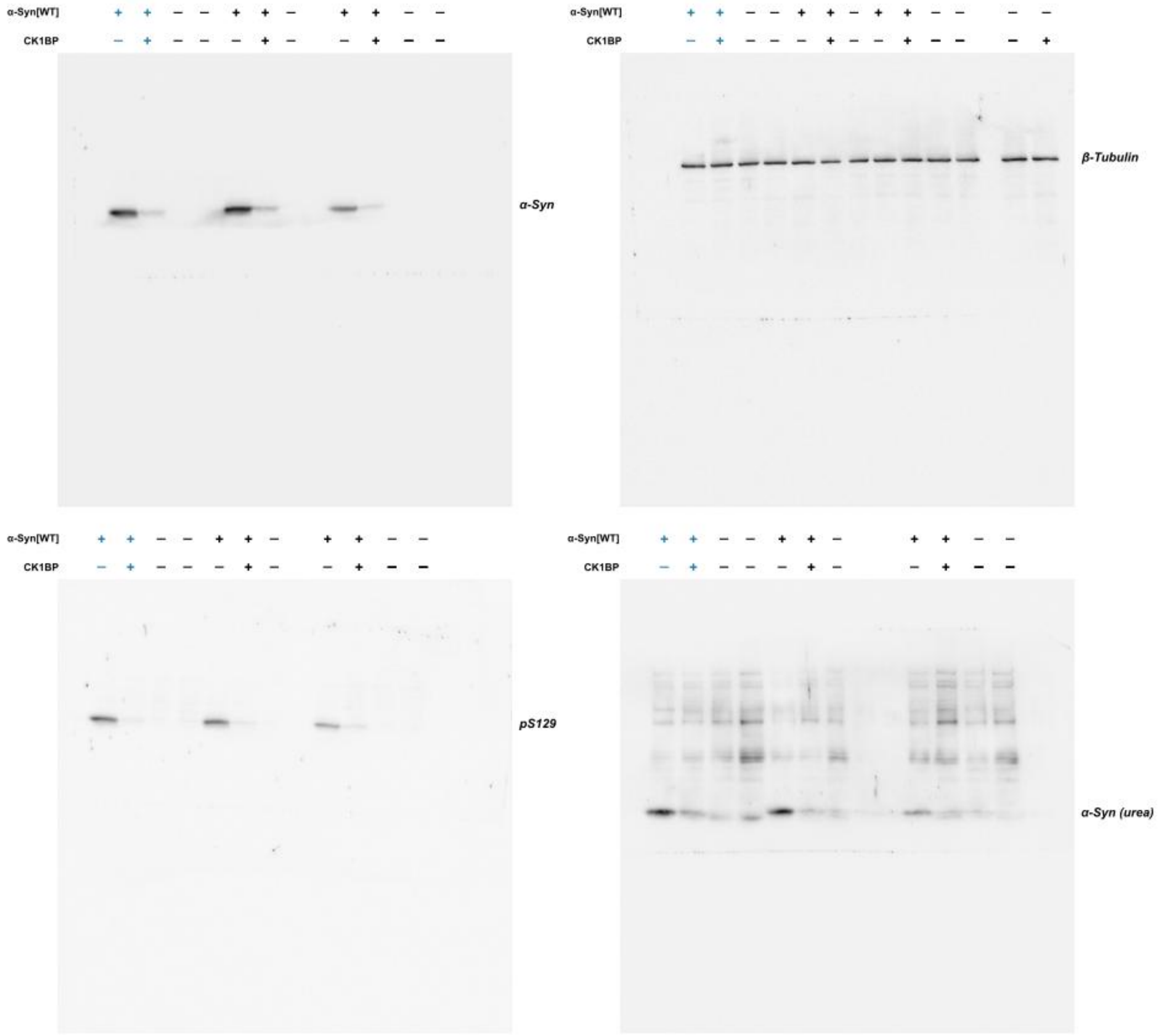

Figure 3D (BiFC-α-Syn[S129D])

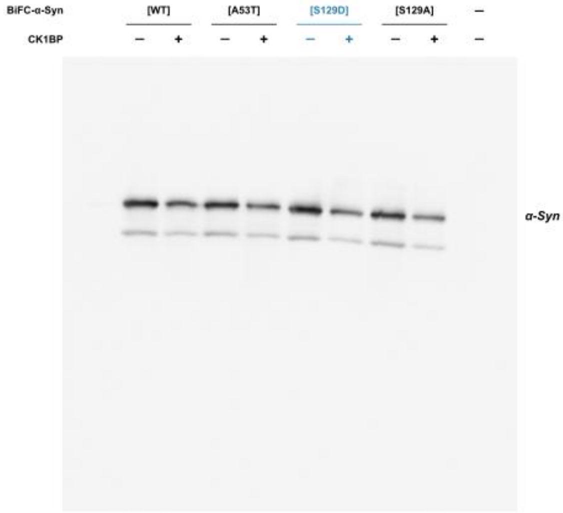

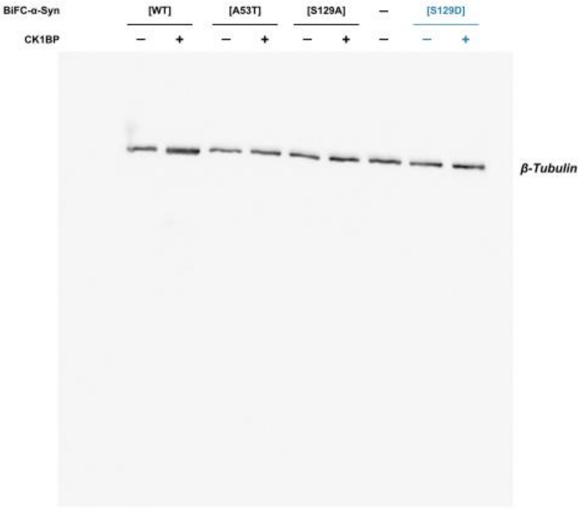

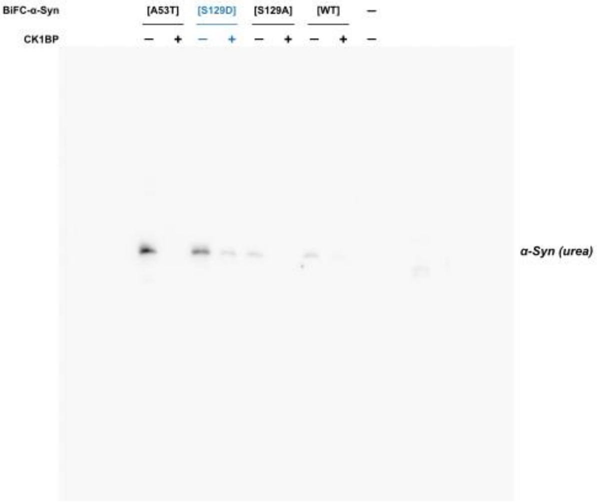

Figure 3D' (BiFC-α-Syn[S129A])

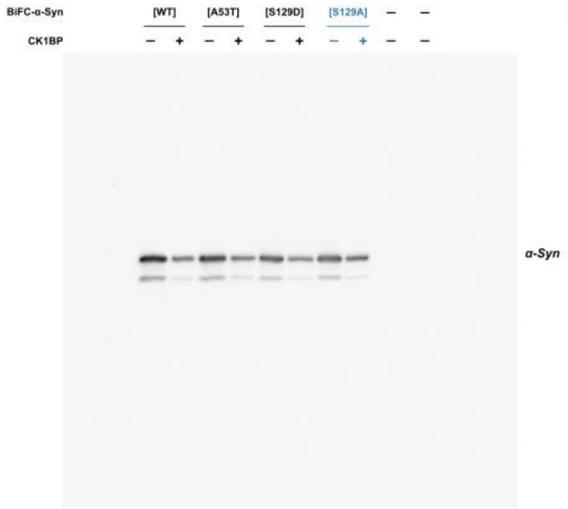

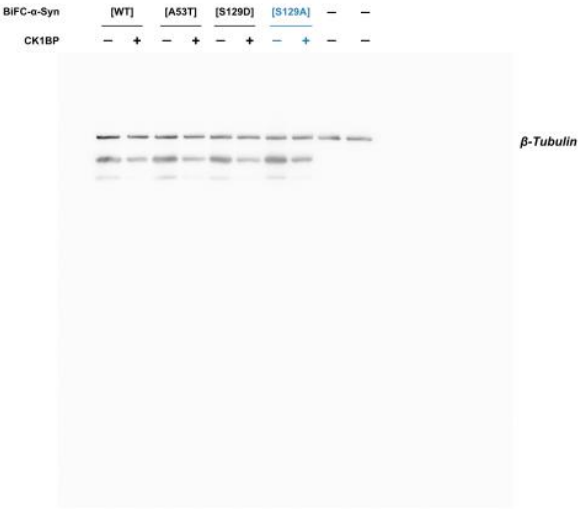

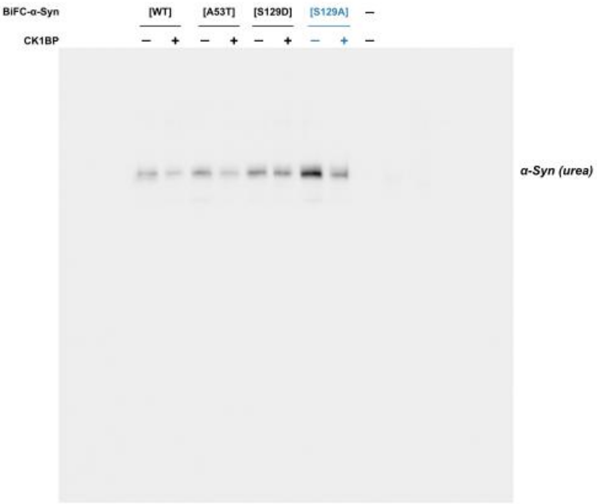

Figure 4E (GFP-polyQ)

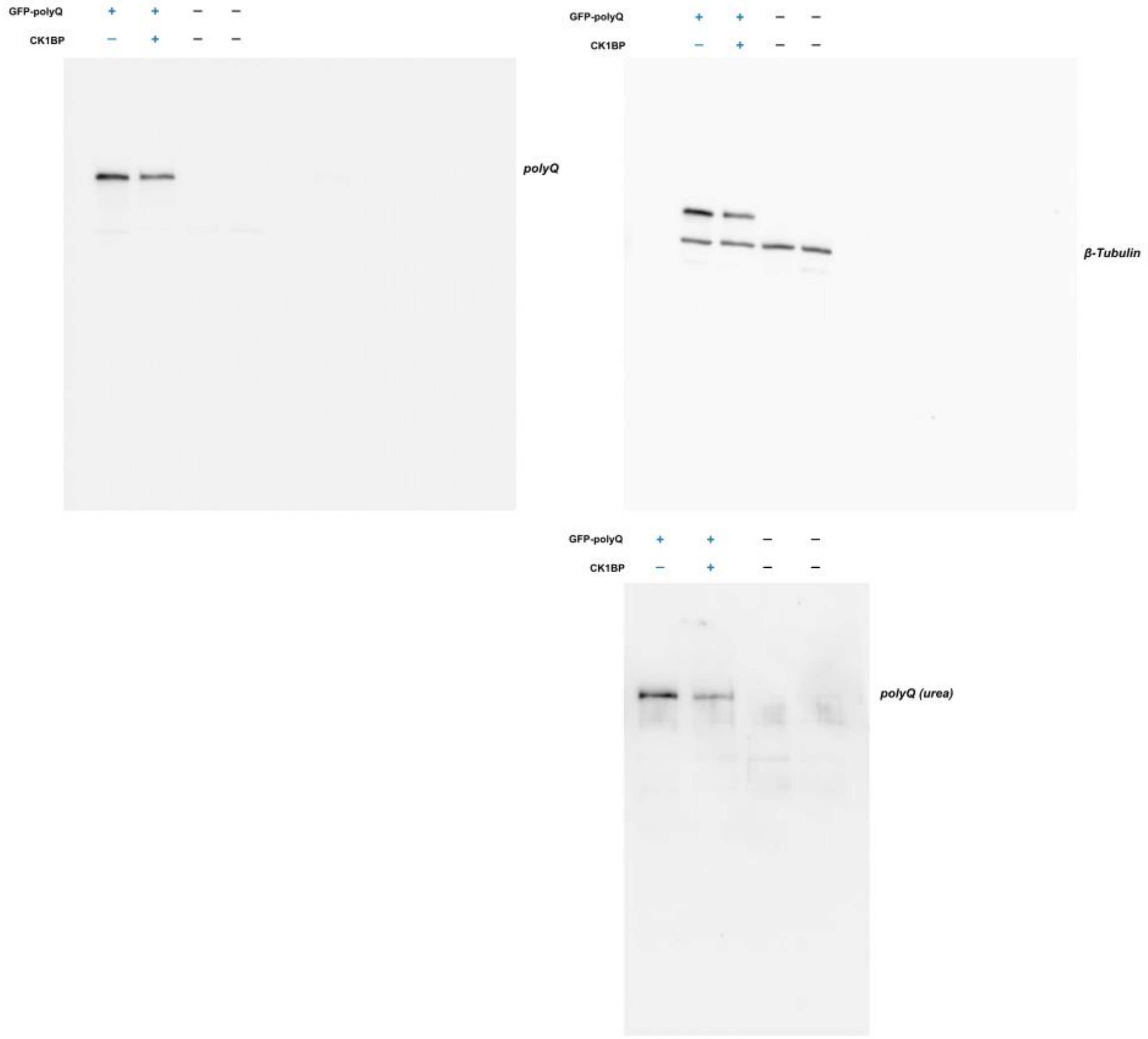

Figure A1B (CK1BP)

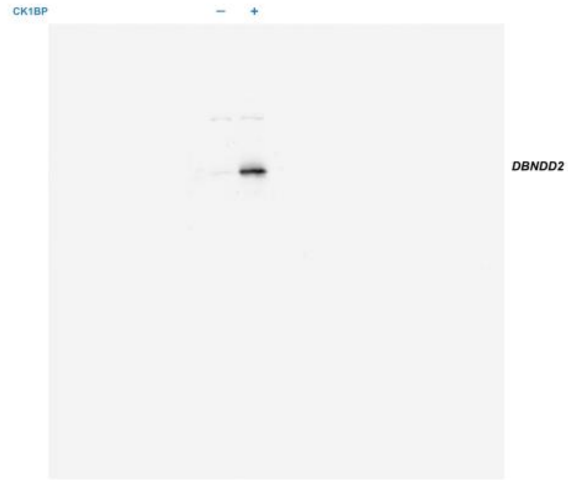

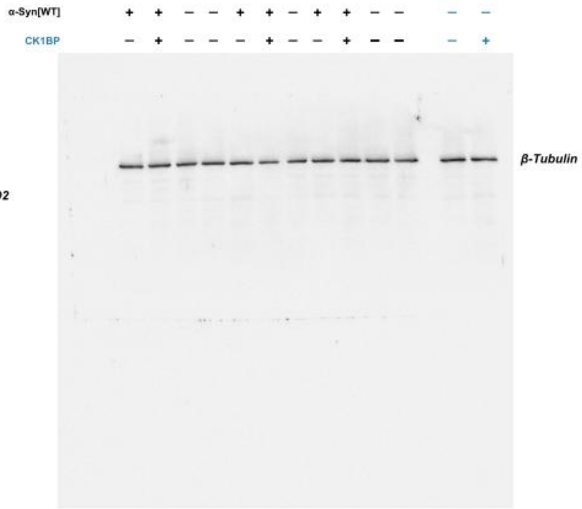

Figure A2C (BiFC-α-Syn[WT])

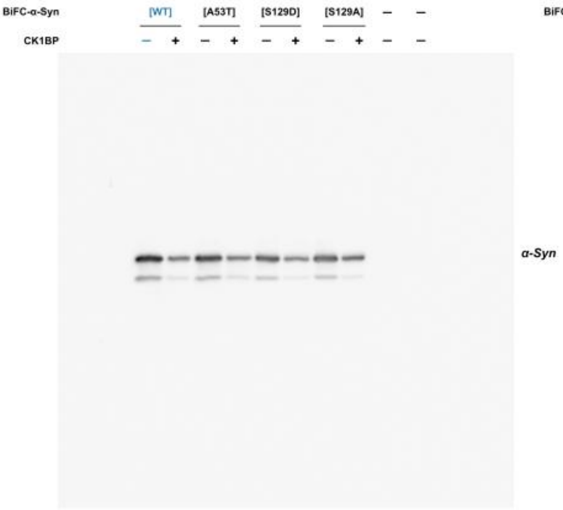

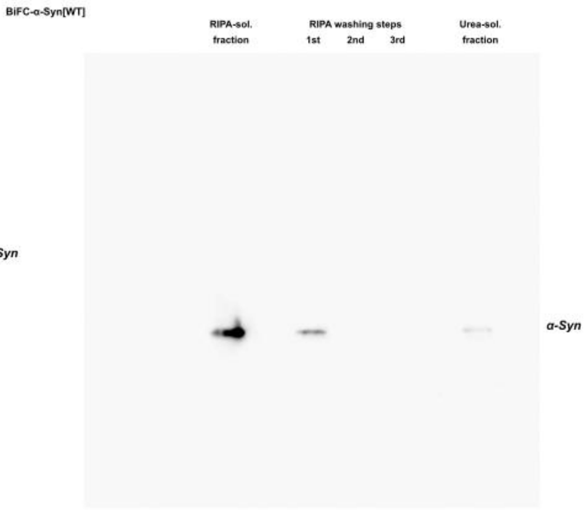

Figure A2C (untagged α-Syn[WT])

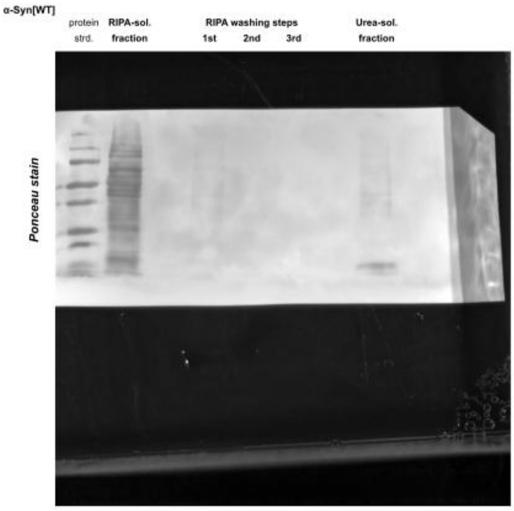

Figure B1E (BiFC-α-Syn)

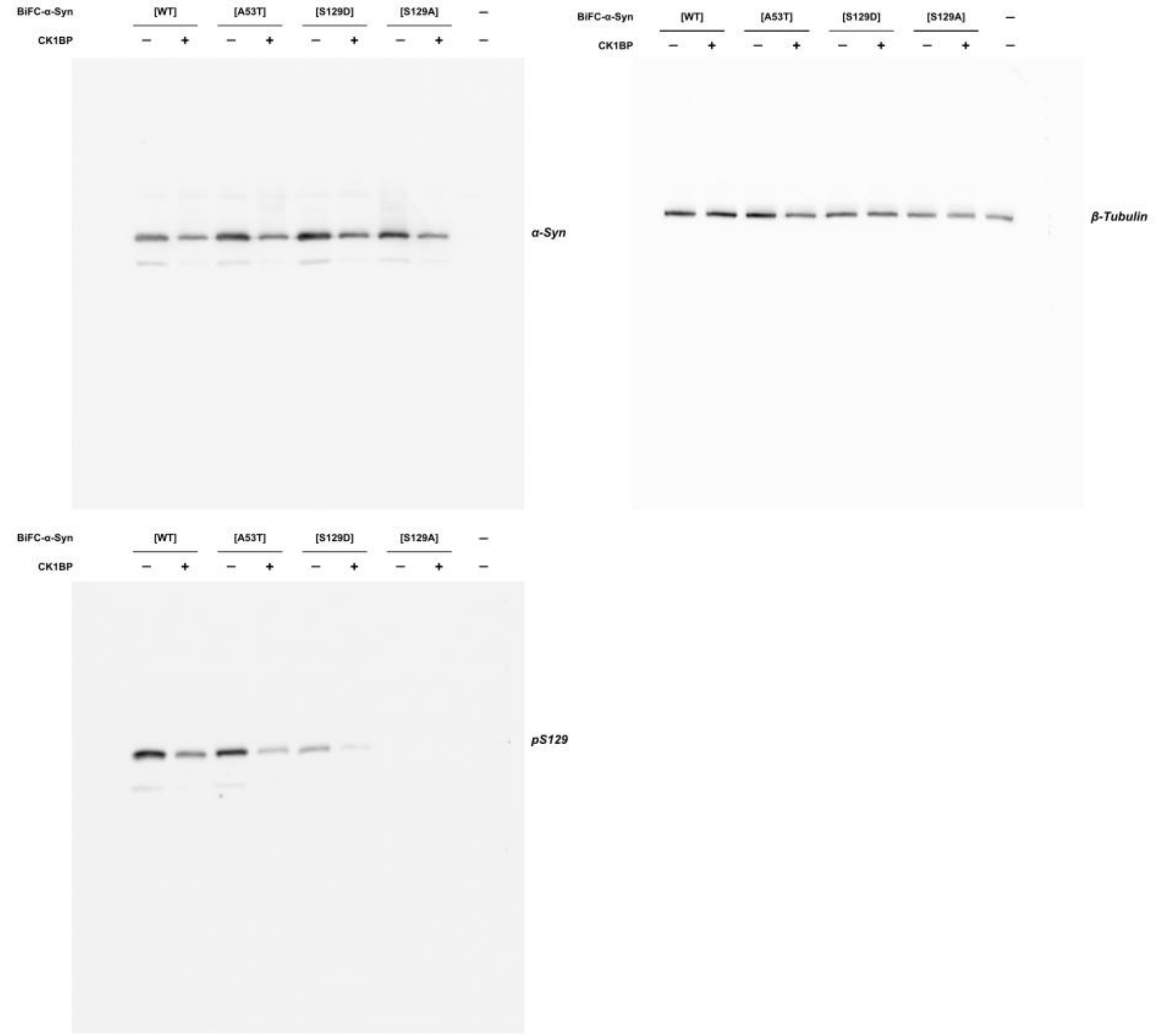

Figure B1F (BiFC-α-Syn[S129A])

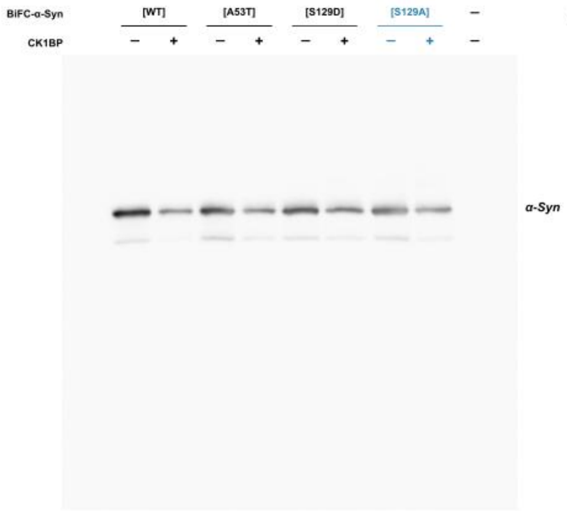

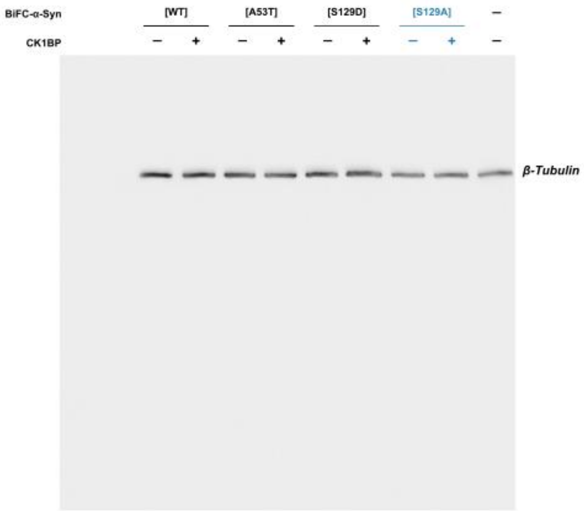

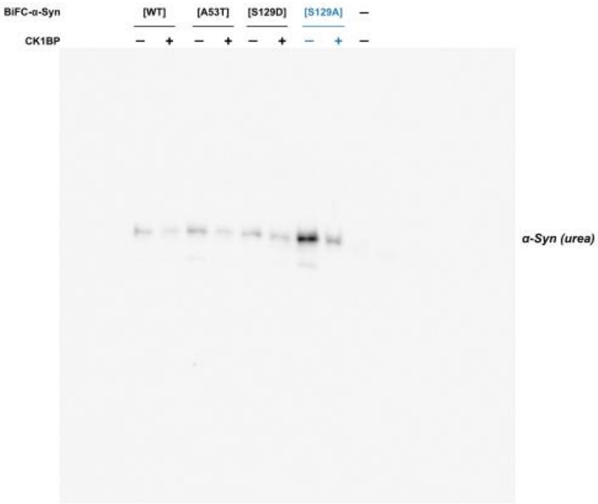

Supplement: Supplementary file 1 [file cells-10-02830-s001.zip › cells-1421786-supplementary.pdf]
